# Engineering Symmetry Breaking Interfaces by Nanoscale Structural-Energetics in Orthorhombic Perovskite Thin Films

**DOI:** 10.1021/acsnano.4c17020

**Published:** 2025-03-06

**Authors:** Duncan T. L. Alexander, Hugo Meley, Michael Marcus Schmitt, Bernat Mundet, Jean-Marc Triscone, Philippe Ghosez, Stefano Gariglio

**Affiliations:** † Electron Spectrometry and Microscopy Laboratory (LSME), Institute of Physics (IPHYS), École Polytechnique Fédérale de Lausanne (EPFL), CH-1015 Lausanne, Switzerland; ‡ Department of Quantum Matter Physics, University of Geneva, CH-1211 Geneva, Switzerland; § Theoretical Materials Physics, Q-MAT, Université de Liège, 4000 Liège, Belgium

**Keywords:** transition metal oxide, orthorhombic perovskite, structural-energetics, interface engineering, switching plane, intermediate layer

## Abstract

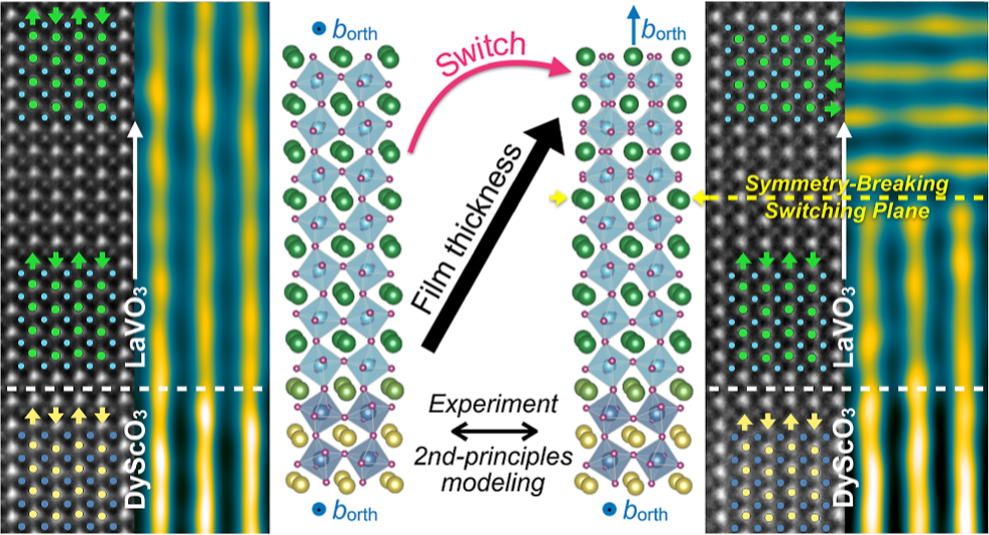

The atomic configuration of phases and their interfaces is fundamental to materials design and engineering. Here, we unveil a transition metal oxide interface, whose formation is driven by energetic influences—epitaxial tensile strain versus oxygen octahedra connectivity—that compete in determining the orientation of an orthorhombic perovskite film. We study this phenomenon in layers of LaVO_3_ grown on (101) DyScO_3_, using atomic-resolution scanning transmission electron microscopy to measure intrinsic markers of orthorhombic symmetry. We identify that the film resolves this energetic conflict by switching its orientation by 90° at an atomically flat plane within its volume, not at the film–substrate interface. At either side of this “switching plane”, characteristic orthorhombic distortions tend to zero to couple mismatched oxygen octahedra rotations. The resulting boundary is highly energetic, which makes it a priori unlikely; by using second-principles atomistic modeling, we show how its formation requires structural relaxation of an entire film grown beyond a critical thickness measuring tens of unit cells. The switching plane breaks the inversion symmetry of the *Pnma* orthorhombic structure, and sharply joins two regions, a thin intermediate layer and the film bulk, that are held under different mechanical strain states. By contacting two distinct phases of one compound that would never otherwise coexist, this alternative type of interface will enable nanoscale engineering of functional systems, such as creating a chemically uniform but magnetically inhomogeneous heterostructure.

Unlike conventional semiconductors, perovskite-structured transition metal oxides (TMO) offer broad possibilities for achieving functional electronic and magnetic properties, by exploiting the correlation between different degrees of freedom (spin, charge, lattice, orbital, topology).^1,2^ Epitaxial perovskite TMO thin-films and heterostructures further promote the coupling of degrees of freedom; on the structural level by setting the strain state via a careful selection of the substrate, as well as on the electronic level by charge transfer or screening effects. The effects of epitaxy have therefore been widely explored, leading to impressive modulation of physical properties, such as the tuning of the metal-to-insulator transition in nickelates,^3^ or of the ferroelectric critical temperature in ferroelectrics.^4^ The success of this type of approach depends on both the sensitivity of the transition metal crystal field to any size modification of the oxygen octahedra, and on the coupling of the transition metal cations via the oxygen anions.^5^

This coupling is founded on the *AB*O_3_ perovskite structure, which can be described by a (pseudo)cubic unit cell of corner-sited transition metal *B*-site cations that each combine with six oxygen anions to form eight corner-shared *B*O_6_ octahedra surrounding a body-centered *A*-site cation;^6^ see Figure 1a. For many TMO perovskites, the *A*-site cation is “undersized” for the space allocated to it. In this case, antiferrodistortive (AFD) tendencies drive the *B*O_6_ oxygen octahedra to undergo rotations which shorten the *A*–O bonds while maintaining the *B*–O distances, thereby increasing energetic stability.^6−10^ The resultant set of oxygen octahedra rotations (OOR) is often described using the Glazer notation, which considers whether the octahedra rotate in-phase (+sign) or out-of-phase (−sign) along the three pseudocubic (“pc”) axes.^11^ In the case of orthorhombic compounds—the most common lattice system of TMO perovskites—the *B*O_6_ octahedra rotate in-phase along one pc axis and out-of-phase along the other two; see Figure 1b. Using the conventional *Pnma* space group setting, the in-phase axis corresponds to the long orthorhombic axis *b*_orth_ having lattice parameter ≈ 2*a*_pc_ (where *a*_pc_ is the lattice parameter of the pc unit cell), while . (Note that, to transform to the commonly used *Pbnm* setting, the *Pnma* axes *a*, *b*, *c* are reordered to *c*, *a*, *b*.) An extra component of energetic stabilization comes from the condensation of antipolar displacements of the *A*-site cations.^6,9,10,12,13^ Deriving from other AFD instabilities, these are energetically coupled to the OOR by a trilinear term.^9,10^ The more significant is the *X*_5_^–^ mode, involving positive and negative displacements of *A*-site cations along the *a*_orth_ axis when following a lattice vector parallel to *b*_orth_;^14^ see Supporting Information Figure S1. This mode itself constitutes a unique signature of the orthorhombic *a*^–^*b*^+^*c*^–^ OOR pattern.^15^

**Figure 1:**
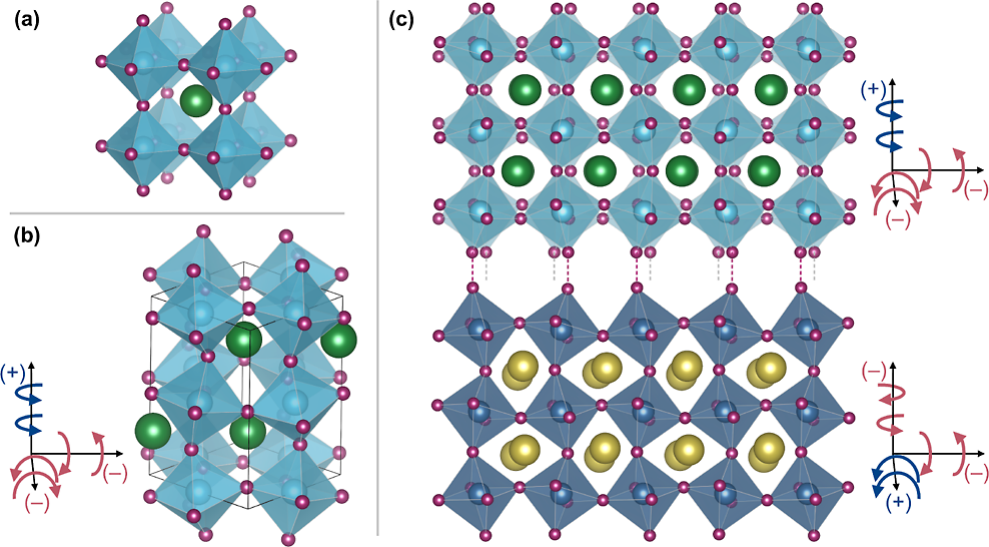
Illustration of structural conflict at the rotation-coupled interface. Panel (a) shows the cubic perovskite unit cell, with six oxygen anions (purple spheres) forming an octahedron centered around each corner-sited *B* cation (blue spheres). The *A* cation (green sphere) sits in the middle of eight *B*O_6_ octahedra. The orthorhombic unit cell variant of the perovskite structure is shown in (b), with the distortions and related out-of-phase (−) and in-phase (+) *B*O_6_ rotation axes of its *Pnma* symmetry. Panel (c) depicts the meeting of two *Pnma* structures, the lower one having the in-phase OOR axis in the substrate plane, while it is perpendicular to the substrate plane in the upper one. By looking along a projection parallel to the in-phase OOR axis of the lower structure, we see that, at the interface of the two, only half of the apical oxygens of the upper structure can match directly to those of the lower structure (purple dashed lines). The remaining apical oxygens cannot form a match across the interface (gray dashed lines). Structural models prepared with the aid of VESTA.^24^

A key parameter determining the electronic coupling of the transition metal cations is the *B*–O–*B* angle. Since this in turn derives from the rotations of the corner-connected *B*O_6_ octahedra, the connectivity of OOR across heterostructure interfaces presents an effective route toward controlling the *B*–O–*B* angle.^16^ Moreover, aberration-corrected scanning transmission electron microscopy (STEM) has revealed that the OOR of a substrate or buffer layer can create an imprint on the OOR of a thin film, with an out-of-plane extent that depends on the symmetries either side of the epitaxial interface (cubic/orthorhombic, orthorhombic/orthorhombic), and on the rotation amplitudes of the materials.^17,18^

Besides OOR connectivity, epitaxial strain state is the main driver determining orthorhombic film orientation. In *Pnma* perovskites, the *B*–*B* distance is shorter parallel to *b*_orth_ than along the two pc axes perpendicular to *b*_orth_. Therefore, if a film is grown under biaxial epitaxial tension, the resulting tensile strain is lower when *b*_orth_ orients out-of-plane (see Supporting Information Table S1 for the system studied here). Hence, in the absence of strain-relieving defects, the film’s macroscopic strain energy is minimized by *b*_orth_ orienting out-of-plane.^15,19−23^ (As caveat are certain cases on cubic substrates where pc shear strain also plays a role.^15^)

The *b*_orth_ axis is the in-phase OOR axis of the film; if it orients out-of-plane, the two pc axes with out-of-phase OOR therefore orient in-plane. However, if a *Pnma* (101)_orth_ orthorhombic substrate is selected, it instead has *b*_orth_ in-plane. As shown schematically in Figure 1c, along substrate *b*_orth_, there is consequently a mismatch of the substrate’s in-phase OOR with the out-of-phase OOR of the tensile strain state-favored film. This mismatch sets up a structural-energetic conflict, with the interface favoring an alternative film orientation, as detailed in the next section. Here, we unveil how the system resolves this conflict by creating a coherent structural interface that we term the switching plane. Formed within a chemically uniform TMO film, this corresponds to an atomically thin boundary where its *Pnma* structure switches orientation by 90°, thereby breaking the inversion symmetry of the orthorhombic perovskite lattice. At the same time, through detailed STEM characterization of layers of LaVO_3_ deposited on (101)_orth_ DyScO_3_, complemented by innovative second-principles modeling, we develop deep insights into the nanoscale structural-energetics of the orthorhombic film growth, and how the final atomic structure of the film depends on film thickness. As described later, these findings open unique opportunities for the deterministic engineering of novel functional properties.

## Results and Discussion

### Film Nanostructure Analyses

**Figure 2:**
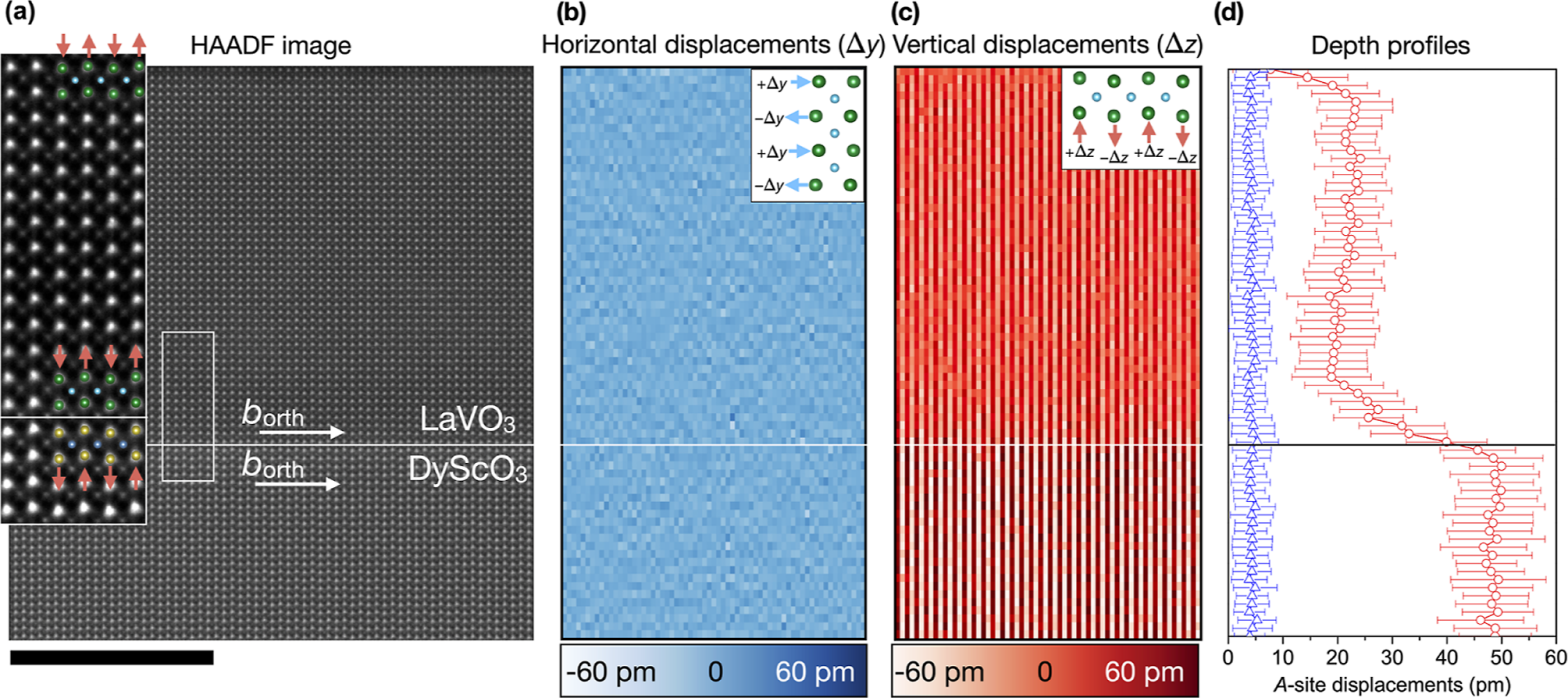
STEM analysis of the 52 uc LaVO_3_ on DyScO_3_ heterostructure viewed along the [100]_pc_/[101̅]_orth_ substrate zone axis. Panel (a) shows a HAADF image from the substrate up to the film surface, with the inset presenting a zoom of the region at the film–substrate interface indicated by the white rectangle. The OOR patterns of the film and substrate are revealed through the observation of the projected *X*_5_^–^ AM mode; the up–down displacement of successive Dy planes when moving along a left–right direction in the substrate propagates into the La sites across the whole film thickness. This qualitative impression is confirmed by a quantification of the *A*-site cation positions for (b) horizontal Δ*y* displacements and (c) vertical Δ*z* displacements. (Note that these two panels are compressed on the horizontal axis.) The average of the magnitude (i.e., modulus) of the Δ*y* and Δ*z* displacement values along a depth profile are shown in panel (d), with the curves color-coded according to panels (b) and (c). Only a vertical displacement of the *A*-site cations is seen, confirming that *b*_orth_ remains in plane across the whole film thickness. In the film, there is a gentle decay in the magnitude of the AM over the first ∼8 uc from the interface, from the substrate value to that of the “bulk film”. On this plot the error bars represent the measurement standard deviation. Scale bar: 10 nm.

To develop a basis for understanding switching plane formation, in Figure 2 we first explore the consequence of OOR mismatch on a 52 pc unit cell (uc) thick LaVO_3_ film grown, by pulsed laser deposition, under ∼0.5% biaxial tension on a (101)_orth_ DyScO_3_ substrate (see Supporting Information Table S1 for full strain state calculations). Figure 2a shows a STEM image of the sample, recorded on the [100]_pc_/[101̅]_orth_ zone axis of the substrate. Using the atomic number contrast of the high-angle annular dark-field (HAADF) detector, it depicts the brighter *A*-site and darker *B*-site cations. This enables imaging of the *X*_5_^–^ antipolar motion (AM) of the *A*-site cations (Supporting Information Figure S1). Since, in the substrate, *b*_orth_ lies in-plane, in Figure 2a its *X*_5_^–^ mode is seen in projection as a vertical AM of successive layers of *A*-site cations. We emphasize that, because of the trilinear energetic term coupling AM with the *a*^–^*b*^+^*c*^–^ OOR, visualization of this mode can be used as a proxy for determining the in-phase OOR axis and relative amplitude.^21,25−27^Figure 2a shows that the up–down AM propagates across the entire LaVO_3_ film. Evidently, the energetic cost of coupling the mismatched OOR of the strain-state favored film with those of the substrate is sufficiently high that the film has instead adopted the symmetry and *B*O_6_ rotation configuration of the substrate, keeping *b*_orth_ in-plane. As confirmation, quantified maps of the *A*-site cation positions in Figure 2b,c show that their projected AM remains purely in the out-of-plane direction across the film. The quantified line profile Figure 2d in turn shows that the magnitude of displacement decays over ∼8 uc from substrate into film, while the continuity of in-phase OOR from substrate into film is confirmed by annular bright-field (ABF) STEM imaging around the film–substrate interface (Supporting Information Figure S2).

**Figure 3:**
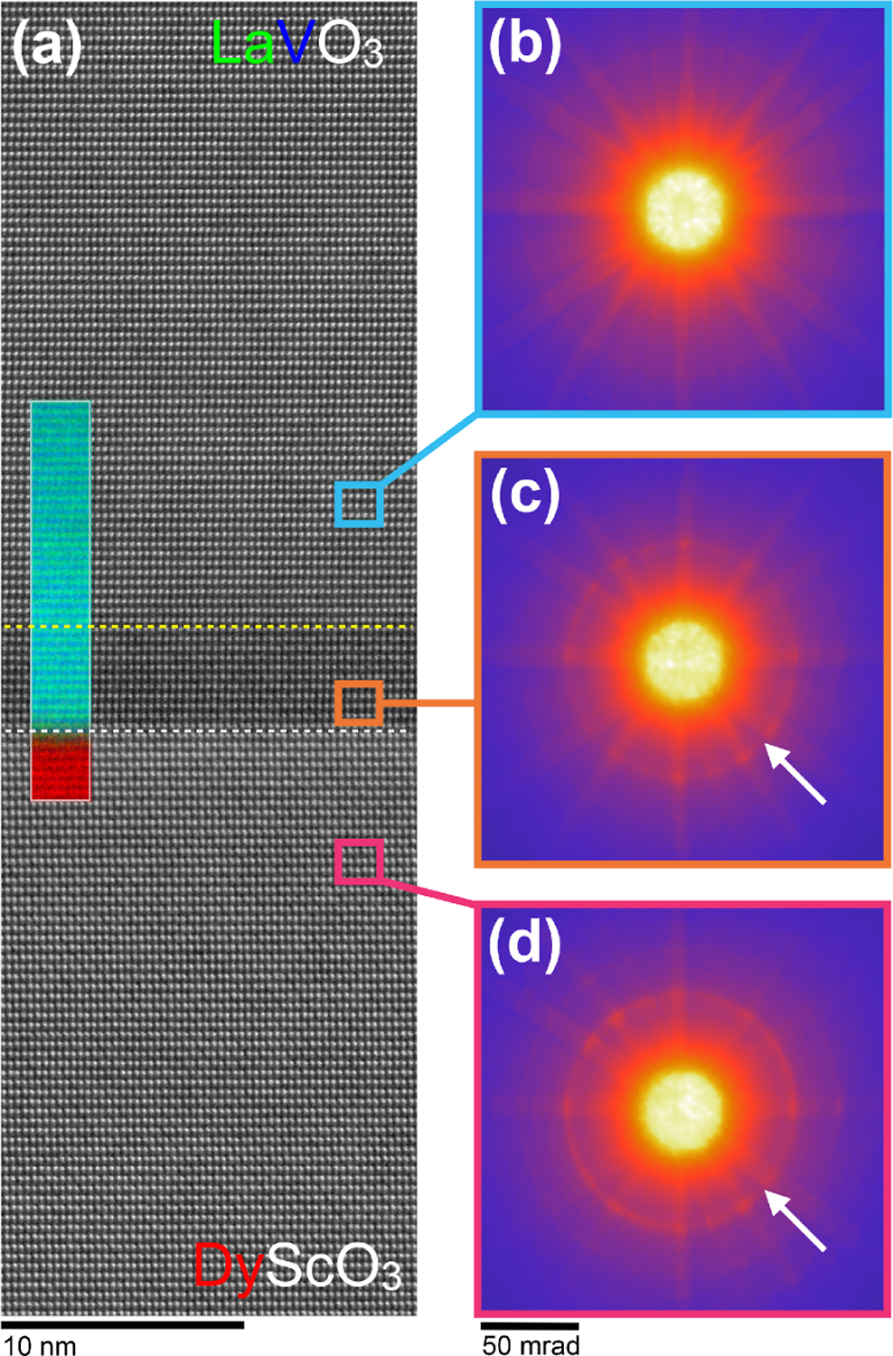
(a) STEM-HAADF image of lower part of ∼110 uc LaVO_3_ film on DyScO_3_, recorded on the [110]_pc_ zone axis using a large inner collection semiangle of ∼80 mrad. The white dashed line indicates the film–substrate interface. Remarkably, the first 10 uc of the film up to the yellow dashed line show a darker contrast than the bulk, even though the film composition is uniform, as illustrated by the overlaid EELS map for La (green), V (blue) and Dy (red). (b–d) PACBED patterns taken from the same film and zone axis at the analogous indicated positions in the substrate and film. The arrow in pattern (d) indicates a low angle first order Laue zone (FOLZ) ring, from the substrate having a [111̅]_orth_ zone axis. This FOLZ ring is maintained in pattern (c) that is taken from the region of film having a darker contrast. However, the ring disappears in pattern (b) for the film bulk, that in turn has a [001]_orth_ zone axis.

Similar imposition of substrate symmetry and *B*O_6_ rotations has been systematically observed, in analogous *Pnma*/*Pnma* film/substrate combinations, for films up to 35–40 uc thick; an outcome that holds even for tensile strains >2%.^15,18,20,28−32^ In line with these previous reports, we observe that the film is monodomain, because its orientation is set by a single crystal substrate. Nevertheless, the film has an increased macroscopic strain energy over one having *b*_orth_ out-of-plane; a strain energy that scales with film thickness. Therefore, one may ask, if film growth is continued, is there a critical thickness at which an epitaxial orientation transition will be induced? That is, a thickness at which the energetic gain from the film adopting *b*_orth_ out-of-plane is enough to force the creation of an interface that couples the mismatched OOR of substrate and bulk film? As a first step in looking at this question, Figure 3a shows a lower magnification HAADF STEM image of a much thicker LaVO_3_ film. As established before,^21^ the bulk lattice orientation of this film actually is determined by the biaxial tensile strain, with *b*_orth_ out-of-plane. Structurally, therefore, the mismatched OOR of bulk film and substrate must have been accommodated. This image, recorded on the [110]_pc_ ([111̅]_orth_ DyScO_3_) zone axis, provides a first hint at this accommodation, from a band of darker contrast ∼10 uc thick that lies within the film, running along the film–substrate interface. Compositional analysis finds that this darker contrast is not chemical in origin (Figure 3a, Supporting Information Figures S3 and S4). It is instead structural, as indicated by the accompanying position averaged convergent beam electron diffraction (PACBED) patterns of Figure 3b–d.

**Figure 4:**
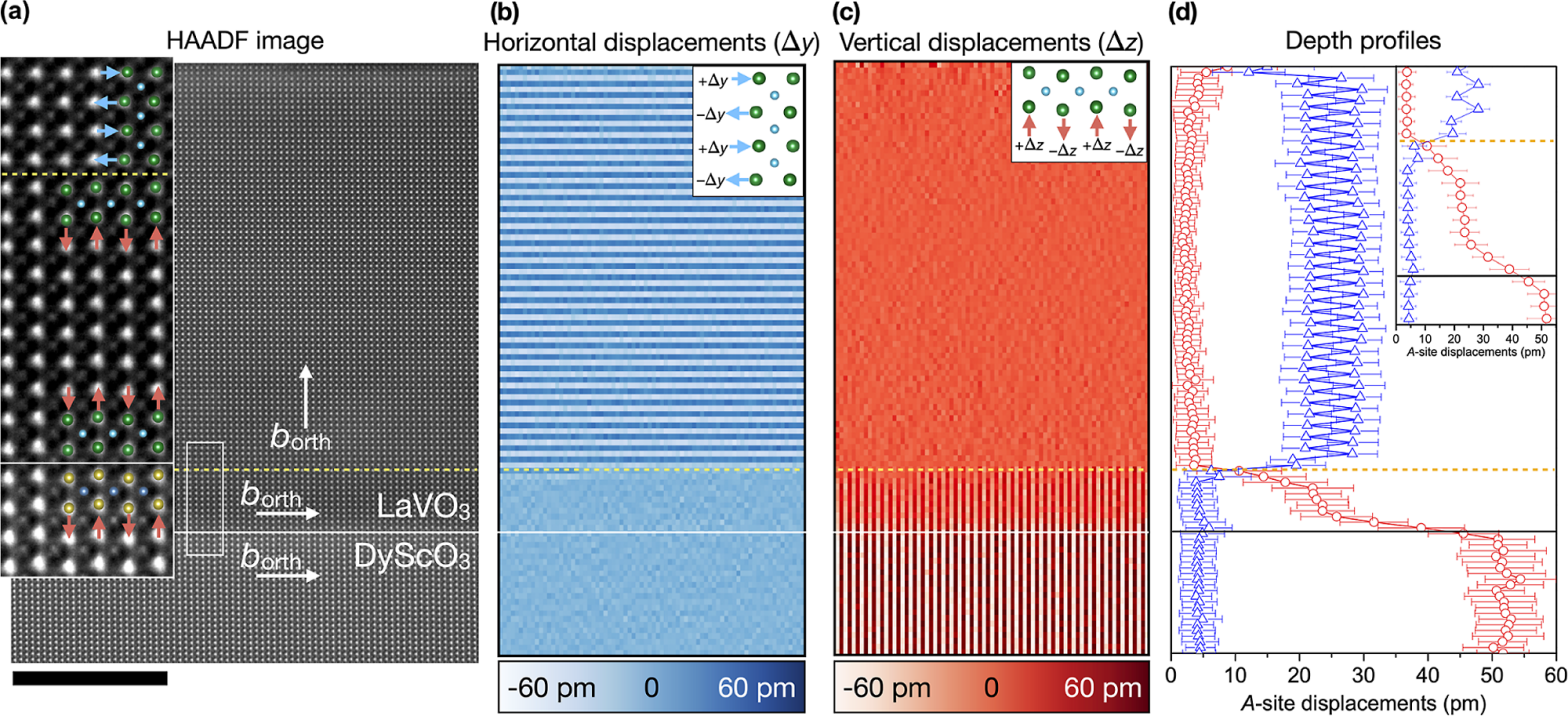
STEM analysis of 81 uc LaVO_3_ film grown on DyScO_3_ viewed along the [100]_pc_/[101̅]_orth_ substrate zone axis. (a) HAADF image across the full thickness of the film, with the inset showing a zoom of the region at the film–substrate interface indicated by the white rectangle. Based on this image, panels (b–d) present quantified analyses of the *A*-site cation positions similar to those made in Figure 2. From this data, it is seen that the substrate’s *X*_5_^–^ AM mode propagates into the LaVO_3_ over ∼10 uc. At the 11th uc, the vertical AM mode abruptly decays to zero and then switches to a left–right displacement of successive La planes. This change in the orientation of the projected *X*_5_^–^ AM mode corresponds to a reorientation of the *Pnma* unit cell to having *b*_orth_ perpendicular to the substrate plane—as favored by the biaxial strain state for the LaVO_3_ film. The plots (b,c) (compressed on the horizontal axis) and (d) prove that this switch is abrupt, going from projected displacements that are purely vertical to purely horizontal at a well-defined distance from the film–substrate interface, as indicated by the yellow dashed line. Indeed, the single uc shift in the position of this “switching plane” visible toward the left of the plot (b) mirrors a step edge in the substrate. The inset of plot (d) focuses on the region of film between the film–substrate and switching plane interfaces. In this intermediate layer, it is seen that the magnitude of AM decays rapidly from the substrate value to a plateau ∼5 uc in length, before decaying sharply to zero over the last few unit cells. On this plot the error bars represent the measurement standard deviation. Scale bar: 10 nm.

We now study this structural formation in more detail, in an 81 uc thick film. We use HAADF STEM imaging on the [100]_pc_/[101̅]_orth_ zone axis of the substrate to evaluate the *A*-site AM of the orthorhombic lattices. Compared to the alternative approach of “direct” imaging of in-phase *B*O_6_ rotations using, for instance, ABF, this enables measurement of the orthorhombic distortions of *b*_orth_ both in-plane and out-of-plane in a single image. It is further robust to residual aberrations and sample mis-tilts, such that quantitative atomic data can be taken across the entire ∼35 nm thickness of the film, with a 4k × 4k pixel resolution. Figure 4a presents an example image. In the DyScO_3_ substrate at the bottom of the zoomed inset, the *A*-site cations have an up–down AM for *b*_orth_ in-plane. In contrast, higher up in the LaVO_3_ film they displace left–right, indicating that it has adopted *b*_orth_ out-of-plane, as favored by tensile strain. Surprisingly, however, the transition between these two orientations does not occur at the film–substrate interface. Instead, the AM of the substrate propagates into the film over ∼10 uc, and only then switches orientation. In Figure 4b,c, this observation is studied with quantified maps of *A*-site left–right (Δ*y*) and up–down (Δ*z*) displacements. In a segment that we term the intermediate layer (IL), the AM of the substrate continues into the LaVO_3_, before a sharp segue to the left–right AM of the film bulk. Figure 4d shows depth profiles of the average *A*-site displacements across the IL. Going from the substrate into the thin film, the projected AM displacements remain purely parallel to the pc *z*-axis. At the same time, their amplitude decays over a few unit cells to a plateau of ∼5 uc length. At the end of this, the amplitude further decays rapidly, reaching a value near zero at the top of the IL. At this point—which we term the switching plane—projected AM displacements switch sharply to being parallel to the pc *y*-axis, corresponding to an out-of-plane *b*_orth_. The displacements then increase in amplitude over a few uc to reach their final value for the bulk film structure. The amplitude of AM in the IL plateau region is approximately equal to that of the film’s bulk structure. In agreement with this analysis, ABF STEM along substrate [010]_orth_ shows directly how the in-phase OOR of the IL tend to zero at the switching plane (Supporting Information Figure S5).

The initial continuity of substrate symmetry into the film over some 10 uc, followed by a switch to *b*_orth_ out-of-plane, explains the PACBED results along [110]_pc_ in Figure 3. The corresponding [111̅]_orth_ zone axis of the DyScO_3_ substrate gives it a strong first order Laue zone (FOLZ) ring at a low scattering angle of ∼60 mrad, owing to a symmetry distance doubling along the beam path direction.^33^ Because of the structural continuity, this FOLZ ring remains in the IL; however, it disappears in the film bulk when the zone axis switches to [001]_orth_. 4D-STEM proves that this disappearance occurs discretely, when stepping one uc across the switching plane (Supporting Information Figure S6). As discussed in Supporting Information Figure S7 comparing the LaVO_3_ bulk and DyScO_3_ substrate PACBED patterns to their simulated counterparts, intensity peaks in the DyScO_3_ FOLZ ring relate to *A*-site antipolar displacements.^34^ Because of the symmetry continuity, these intensity patterns remain in the FOLZ when moving into and across the IL. Together with simulations using μSTEM,^35,36^ the PACBED analysis also explains the darker contrast from the IL in Figure 3a. Specifically, this arises from strong elastic scattering into the FOLZ rings of the substrate and IL that consequently leads to them having anomalously low thermal diffuse scattering and hence decreased signal on the HAADF detector (Supporting Information Figures S8 and S9). As a result, the dark contrast along the [110]_pc_ zone axis itself constitutes an indication of IL/switching plane formation.

As for a film grown below critical thickness, the IL is monodomain. The film bulk having *b*_orth_ out-of-plane can instead adopt two possible orientations, with either *a*_orth_∥[110]_pc_ and *c*_orth_∥[1̅10]_pc_ or vice versa. These domains can be discriminated on the [110]_pc_ zone axis, where the *A*-site cations show a pure *X*_5_^–^ AM when viewing on [001]_orth_, or instead the subtle, in–out *R*_4_^–^ AM when viewing on [100]_orth_.^14^ (They can equally be discriminated by their half-order reflections in diffraction.) Supporting Information Figures S10 and S11 show HAADF STEM images of the switch from the IL to either of these configurations, together with *A*-site ϵ_*yx*_ strain analysis to help identify the nature of the *A*-site antipolar modes. The domains are large compared to the film thickness, with widths of ∼100–500 nm in cross-section TEM lamellae, as evaluated by large field-of-view atomic-resolution STEM and nanobeam diffraction.

To summarize on this analysis, thicker LaVO_3_ films adopt the orientation expected from the substrate-imposed tensile strain, except for an initial IL which keeps the substrate orientation. The switch between the two orientations occurs sharply, at the switching plane. As described later, the local atomic topography at the switching plane enables a subtle transition between the mismatched OOR of the bulk film and substrate orientations. Associated with this, incompatible orthorhombic distortions tend to a value of zero, while compatible ones propagate freely. Since it contains strong local distortions compared to the *Pnma* structure that forms the basis of the LaVO_3_ lattice, this novel interface is a highly energetic boundary.

Our findings have various implications. First, the critical thickness for transitioning to the strain-state determined bulk film orientation, tied to switching plane and IL formation, is clearly between 52 and 81 uc. By using X-ray diffraction to monitor the appearance of a half-order reflection for *b*_orth_ out-of-plane in a film thickness series, this value is further narrowed to between 60 and 74 uc (Supporting Information Figure S12). Second, given that, during deposition, the film initially grows in structural continuity with the substrate, its atomic lattice must dynamically restructure after reaching the critical thickness, when the bulk film switches its orientation. As discussed later, the restructuring mechanism remains an open question. Finally, we hypothesize that the switching plane combined with IL represent an energetic minimum, as compared to forming an OOR-coupled interface that coincides with the film–substrate interface. This reminds of the stand-off effect of misfit dislocations, where the origin of the strain-releasing defects sits in the elastically weaker material, a few uc distance from the layer–substrate interface.^37−39^ Given its importance, we now explore the energetics of the system via second-principles calculations.

### Second-Principles Calculations

Making appropriate simulations is a nontrivial task, owing to the need to include tens of uc thickness in the film. The “standard” approach of density functional theory (DFT) calculations, as for instance used to calculate the biaxial strain state determined LaVO_3_ structure,^21^ is therefore hardly viable. In order to address this challenge, we innovate a second-principles modeling approach.^40,41^

Building second-principles effective atomic potentials remains very challenging and a substantial work (both for computing an extensive training set of first-principles data and for fully validating the model), which explains why only few high-quality models are presently available. For our simulations, we turn to a system of CaTiO_3_, that we adapt to incorporate physics and constraints analogous to our experimental system. In doing so, we leverage a model that has been previously validated and importantly demonstrated to be accurate for reproducing inhomogeneous structures such as twin walls; see Schmitt,^40^ Zhang et al.,^42^ and Supporting Information Figure S13. Moreover, CaTiO_3_ is the prototypical *Pnma* perovskite, such that our approach implicitly demonstrates the generality of the concept underpinning switching plane formation, that depends on applied constraints rather than specific compound. Finally, CaTiO_3_ is nonmagnetic, aiding tractability of the calculations.

**Figure 5:**
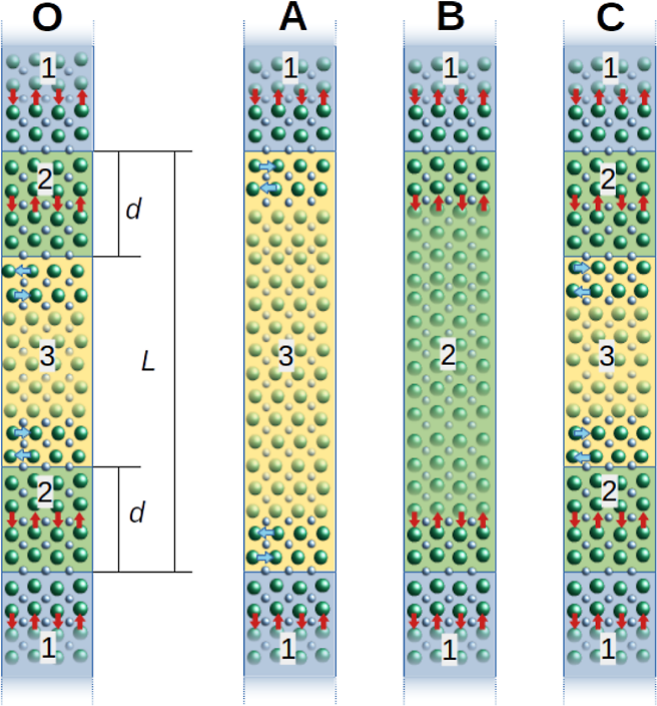
Structural simulations. (O) Illustration of the supercell layout used for the second-principles simulations. The supercells are composed of three parts. Segment 1 mimics the substrate, having *b*_orth_ in-plane but with exaggerated OOR and AM. Its atomic positions are fixed during the relaxation. Segments 2 and 3 mimic the film, with segment 2 having *b*_orth_ in-plane, compared to it being out-of-plane for segment 3. During the simulation, their atomic positions are free to relax. We denote *L* for the total number of uc layers in the film and *d* for the number of uc layers in segment 2. Periodic boundary conditions apply. Three distinct configurations for the film are possible: (A) the whole film consists of *b*_orth_ out-of-plane; (B) the whole film consists of *b*_orth_ in-plane; (C) consists of a mix of both orientations with various numbers of uc layers *d* in segment 2.

The atomic structure “O” in Figure 5 defines the basic supercell setup for the simulations. It makes use of periodic boundary conditions that duplicate the interface. Segment 1 mimics the atomic environment created by the substrate: it is a region of *Pnma* structure with *b*_orth_ in-plane, in which the orthorhombic distortions (OOR and *A*-site AM) are artificially amplified and frozen to replicate the distortion mismatch and biaxial strain imposed by the substrate at the LaVO_3_–DyScO_3_ interface. Segments 2 and 3 comprise the thin film. To represent the IL, segment 2 has a *Pnma* structure with *b*_orth_ in-plane, while segment 3 conforms to the *b*_orth_ out-of-plane structure. Simulations are made for a series of values of absolute film thickness *L*, as specified in uc layers. For each *L*, we consider the energies of the three configuration types illustrated in Figure 5: (A) film of energy *E*_A_(*L*) that consists only of *b*_orth_ out-of-plane (*d* = 0); (B) film of energy *E*_B_(*L*) that consists only of *b*_orth_ in-plane (*d* = *L*/2); (C) films of energies *E*_C_(*L*, *d*) containing a mix of both orientations with IL thickness *d* (0 < *d* < *L*/2). Each structure in the sequence of 0 ≤ *d* ≤ *L*/2 is allowed to relax (atomic positions in segments 2 and 3 and cell parameter out-of-plane), after which their energetic values are compared.

**Figure 6:**
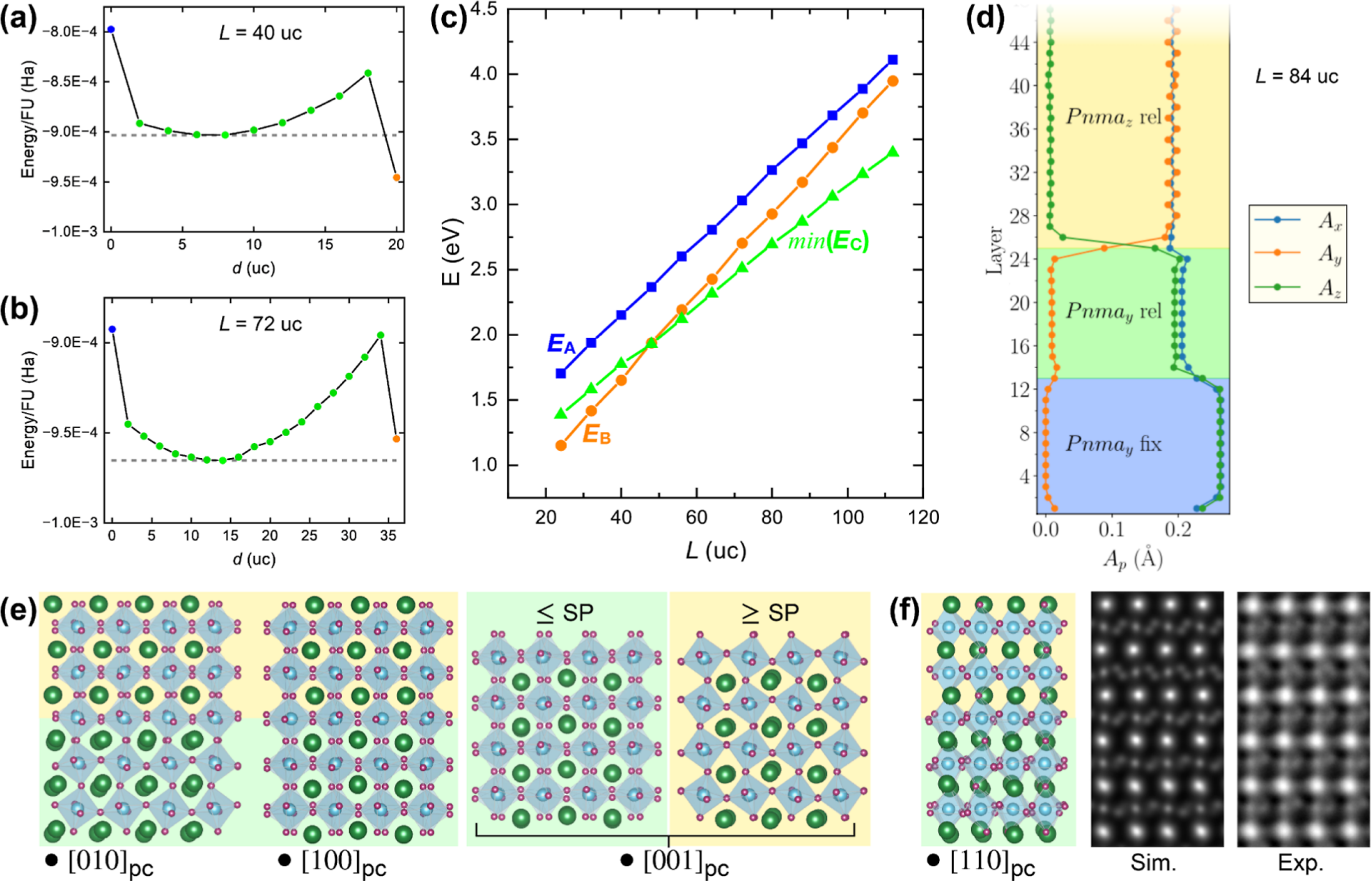
Structure energy as a function of IL thickness *d* for supercells of dimension (a) *L* = 40 uc and (b) *L* = 72 uc. To help compare values, the gray dashed lines indicate the minimum energy for films having configuration C, and the nodes are color-coordinated with plot (c). (c) Relative energies *E*_A_, *E*_B_, and min(*E*_C_) of configurations A, B and C against *L*. The curve min(*E*_C_) represents the minimum possible energy of configuration C, by appropriately optimizing *d* for each *L*. (d) Depth profile of the amplitude of AM displacements of *A*-site cations along the three pc axes across the minimum energy supercell having *L* = 84 uc. Because of the symmetric boundary conditions only the lower half of the supercell is shown. (e) Projections of the *L* = 84 uc minimum energy configuration across the switching plane along different axes, with backgrounds color-coded according to Figure 5; the switching plane is at the junction of the green and yellow sections. (f) Projection along the [110]_pc_ axis with the upper “bulk film” part having a [001]_orth_ zone axis. From this, an inverted ABF STEM image is simulated (substituting Ca and Ti by La and V respectively) and compared to a subframe averaged experimental image from a sample of ∼110 uc LaVO_3_ grown on DyScO_3_.

From the simulation results, Figure 6a presents the energetic evolution of a relatively thin film (*L* = 40), in function of *d*. The highest energy is observed for *d* = 0 (blue node), which corresponds to placing the switching plane at the film–substrate interface (configuration A). As *d* increases, the energy decreases to a minimum at *d* = 8 (green nodes), but then rises again. Finally, for *d* = *L*/2 (orange node), the curve shows a sharp decrease to its lowest energy, making B the most favorable configuration; i.e., the symmetry-imposed structure, as for LaVO_3_ films under critical thickness. Considering the results for a thicker film with *L* = 72 in Figure 6b, a similar curve is seen. However, there is one key difference: the minimum energy in the initial concave (at *d* = 14) is now lower than that of the final, sharp minimum at *d* = *L*/2. Therefore, configuration C having an IL and implicit switching plane is now the most energetically favorable. By repeating the calculations for 24 ≤ *L* ≤ 104 (Supporting Information Figure S14), the summarizing plot in Figure 6c is determined, in which min(*E*_C_) corresponds to the minimum *E*_C_(*L*, *d*) by adjusting *d* to an optimized value *d*_min_ for each *L*. Remarkably, the second-principles simulations reproduce the experimentally observed transition, since the lowest energy configuration changes from configuration B with *b*_orth_ uniformly in-plane to the mixed *b*_orth_ in-plane/out-of-plane configuration C at a critical film thickness of, in this case, *L* ≈ 48 uc.

### Structural-Energetics Analysis

To obtain physical insight into these results, it is instructive to decompose the energies as follows
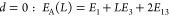
1
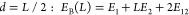
2
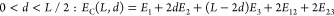
3in which *E*_1_ is the energy of the substrate (segment 1), *E*_2_ and *E*_3_ are the energies per uc of segments 2 and 3, and *E*_*ij*_ are the interface energies between segments *i* and *j*.

In Figure 6c, the energy *E*_A_(*L*) for the *b*_orth_ out-of-plane structure (blue squares) is higher than energy *E*_B_(*L*) for the *b*_orth_ in-plane structure (orange circles) at small thicknesses. This result is driven by the interface energy, with the film preserving the orientation of the substrate to ensure better continuity of the atomic distortions (i.e., *E*_13_ > *E*_23_). However, in terms of elastic energy, the out-of-plane structure is favored (i.e., *E*_3_ is lower than *E*_2_). This means that both curves have a different slope. Extrapolation implies that they will cross at *L* ≈ 140 uc, when sufficient thickness is achieved for the cost of interface energy to be compensated by the elastic energy gain. We further notice that the curves are almost linear, which means that *E*_13_ and *E*_12_ are almost independent of *L*.

While a crossing from configuration B to A could be in line with usual expectations, our second-principles simulations point out that such a transition will never actually happen. Instead, in agreement with our experimental findings, the film will prefer to switch to the mixed configuration C (green triangles)—even though the latter creates an additional energy cost *E*_23_ for the switching plane interface. At small thicknesses, *E*_B_(*L*) < *E*_C_(*L*) < *E*_A_(*L*), highlighting that *E*_12_ + *E*_23_ < *E*_13_. This confirms that it is energetically more favorable to form the OOR-coupled interface within the film, rather than directly at the film–substrate interface. Nevertheless, this alone is insufficient to explain the observed behavior in both simulations and experiments. If *E*_23_ is assumed to be constant, then the slope of *E*_C_(*L*) would be the same as that of *E*_A_(*L*) and also the system would keep *d* minimum (i.e., *d* = 1) at any *L* (since *E*_2_ > *E*_3_). In contrast, the slope of *E*_C_(*L*) is smaller than that of *E*_A_(*L*) and slightly decreasing with *L*, and both experimentally and numerically *d*_min_ ≫ 1. For, crucially, the switching plane energy *E*_23_ depends on *d* and *L*, and progressively decreases with them. Physically, this is because providing larger thicknesses of film on both sides of the switching plane decreases *E*_23_(*L*, *d*), by better accommodating the disparity of atomic distortions. So, on the one hand, the switching plane wants to move away from the film–substrate interface (and surface) to the interior of the film to decrease *E*_23_(*L*, *d*) while, on the other hand, the film wants to keep *d* as small as possible to minimize the elastic energy. The transition from configuration B to configuration C appears at a critical thickness of the film, at which *d* is large enough for the cost of *E*_23_(*L*, *d*) to become small, while (*L* – *d*) is also sufficiently large for the lowering of elastic energy to compensate the switching plane formation. As such, the critical thickness appears as the result of a very delicate balance between distinct energy contributions, which can hardly be quantified without explicit calculations.

In Figure 6d, depth profiles are plotted of the *A*-site AM amplitudes for the min(*E*_C_) configuration of an example film above the critical thickness. *A*_*y*_ and *A*_*z*_ are respectively equivalent to the Δ*y* and Δ*z* values measured along the [100]_pc_ zone axis in Figures 2 and 4. At the film–substrate interface, the amplitude *A*_*z*_ of the substrate AM propagates into the film, but decays over 2–3 uc to a 10 uc plateau. At the end of the plateau, *A*_*z*_ sharply decays toward zero amplitude at the switching plane. At that point, the amplitude of *A*_*y*_ sharply increases from zero to that of the bulk thin film structure of segment 3. These AM transitions mirror those measured for the LaVO_3_/DyScO_3_ system in Figure 4, vindicating the similarity of model to experiments. Notably, the AM amplitude modulation across the switching plane occurs much more sharply than that at the film–substrate interface in films grown below the critical thickness, where the substrate symmetry is preserved (see Figure 2). This can be understood as the system confining the distorted and highly energetic structure around the switching plane to a small volume for energetic minimization. Not only is this discrete switch between two structural phases experimentally confirmed by the STEM and PACBED measurements of LaVO_3_ on (101)_orth_ DyScO_3_, but it represents a case distinct from an alternative type of energetic conflict that was set up in a system of La_2/3_Ca_1/3_MnO_3_ grown on (1̅21)_orth_ NdGaO_3_, where a smooth modulation of orthorhombic distortions over ∼14 uc was observed.^43^

Interrogation of the structural model, and its comparison to STEM data along different zone axes, allow us to elucidate further the nature of atomic structure transition across the switching plane. Figure 6e shows projections of the structural model on the three pc axes. [010]_pc_ is projected along the *b*_orth_ axis of the substrate, and illustrates how the mismatched OOR of bulk film and substrate interface by a sharp flattening or damping within 2 uc either side of the switching plane. Along [100]_pc_, as well as AM we visualize out-of-phase OOR modes for both substrate and bulk film orientations. These two modes do not face the same connectivity problem and, because of this compatibility, we see that the mode propagates undamped across the switching plane. Experimental ABF STEM data are consistent with this finding; see Supporting Information Figure S15. In order to study OOR connectivity on the out-of-plane [001]_pc_ axis, we present two projected slices, each 2 uc thick: one that includes the switching plane uc and the uc below (≤SP) and the other including the switching plane uc and the uc above (≥SP). It is evident that, on this axis, the OOR mode switches from out-of-phase to in-phase very sharply, with negligible damping. Such an abrupt switching in OOR modes along the out-of-plane axis has previously been seen in rhombohedral/orthorhombic heterostructures,^44^ and occurs because the apical oxygen effectively acts as a free pivot point for out-of-plane rotations. In a cross-section sample, these rotations can be monitored by measuring the symmetry of O dumbbells or clusters imaged on a [110]_pc_ axis.^44^Figure 6f shows the appropriate projection, where the bulk film has a [001]_orth_ zone axis. Above the switching plane, the O dumbbells are mirrored by a horizontal plane—indicative of in-phase OOR along the out-of-plane axis. Below it, as the OOR go to out-of-phase, the O clusters lose this mirror symmetry. From the model, an (inverted) ABF image is simulated (“Sim.”), substituting the Ca and Ti cations by La and V respectively, in order to be more easily compared to the experimental data. The experimental image (“Exp.”) on the right bears a resemblance to the simulation, supporting the simulation-based hypothesis that out-of-plane OOR switch directly from out-of-phase to in-phase across the switching plane.

The second-principles simulations made here calculate the ground state energy. Therefore, they do not inform on how a film restructures from configuration B to configuration C when it reaches the critical thickness. Being captured by our second-principles model, the transformation can be seen as “displacive” (rather than totally reconstructive), in the sense that it preserves bond-topology. Hence, in principle, it just requires enough thermal energy for atoms to hop from their old local energetic minimum to a new one. However, from preliminary results, whether the transformation mechanism is instead more complex or involved, and also when it occurs—during growth or postdeposition cooling—remain questions that need further work to resolve.

## Conclusions and Perspectives

In summary, through experiments and simulations, we have revealed a complex “phase space” for guiding the design choice of orthorhombic film growth, set by factors of epitaxial strain, OOR connectivity and film thickness. As shown by simulations in Figure 6, under our chosen *Pnma*/*Pnma* parameters, films grown below a critical thickness have a structural phase set by OOR connectivity. Above the critical thickness, the film instead adopts a two phase structure: a bulk structure set by epitaxial strain, and a thin IL adjacent to the substrate which instead follows the substrate orientation. The calculations further show that considering only the epitaxial strain in OOR/strain imposed systems^21,43^ gives the highest film energy of all structural variants, and so does not correctly predict the film’s complete atomic structure. Indeed, our simulation approach could also be useful for modeling other systems, such as manganite thin films grown under alternative energetic impositions,^43^ or whose energetics remain unexplained.^45^

In films grown beyond the critical thickness, the switching plane is formed between the two phases of the film. Its formation is driven solely by energetics, with no stochastic role of grain or domain nucleation. One consequence is that it appropriates the atomic flatness of the substrate, as for instance seen by the switching plane mirroring the substrate step edge in Figure 4. Simulations nevertheless imply that the IL thickness *d* derives from a broad energetic minimum (see energetic curves in Figure 6b and Supporting Information Figure S14). The exact IL thickness is therefore expected to be sensitive to subtle factors during switching plane formation. This could explain small differences in IL thickness that are sometimes observed at different sampling points for a single deposited film, and is an aspect we are investigating with further experiments. While the atomic landscape of the IL and switching plane is confined to a relatively short length-scale of some 10–15 uc, it is a consequence of energetics acting over the entire film, grown beyond a significant critical thickness of tens of uc. Indeed, its formation can only be predicted by including the film’s full atomic structure in a simulation. Because of this subtlety, conceivably its presence has been missed in previous work.

In TMO materials, it is widely known that heterostructure interfaces that break the bulk lattice symmetry can be exploited to create functional properties beyond the scope offered by the unbound crystal,^46^ such as the formation of a two-dimensional electron system at the interface between insulating LaAlO_3_ and SrTiO_3_ compounds.^47^ Within single-phase compounds, crystalline boundaries such as domain walls in ferroelectric or ferroelastic materials have themselves produced emergent properties distinct from their bulk counterparts.^48−50^ In comparison to these priors, the 90° orthorhombic structure rotation at the switching plane breaks the inversion symmetry of the lattice, which could lead to the emergence of topological states (i.e., Berry phase).^51^

The switching plane separates two regions of the same chemical compound (IL and film bulk) that are held under distinct mechanical boundary conditions (e.g., Supporting Information Table S1). Because it is induced by simple opposition of energetic influences on epitaxial film growth, and with *Pnma* perovskites sharing similar strain differences for *b*_orth_ out-of-plane or in-plane, it is in principle generic for other *Pnma*/*Pnma* film/substrate combinations. Given that the orthorhombic lattice is the most common perovskite structure,^11^ this gives a wide potential for creating the IL and switching plane in other compounds, with a primary requirement of achieving film growth in the absence of strain-relieving defects. Some interesting candidates are manganite, ruthenate, or nickelate films. In these, we can exploit the different strain conditions of the IL and bulk film to engineer adjacent regions that, despite being of the same compound, have distinct electronic and magnetic properties, with the switching plane forming an unprecedentedly sharp interface between them. The IL of such a structure could, for instance, be used to pin the magnetic phase in a LaMnO_3_ film grown on GdScO_3_ to the *b*_orth_ reorientation, thus forming a chemically uniform material that is magnetically inhomogeneous.^14^ Other possibilities for engineering novel functional properties also open up, such as leveraging this atomically flat interface to create a 2-dimensional conductor.^52^ Finally, the control of the crystallographic orientation of the orthorhombic layer provided by the strong interfacial coupling of the OOR, that dominates over the lattice strain up to a critical thickness, offers a way to engineer complex heterostructures where the functional properties, for instance the magnetization axis of ruthenate compounds,^53,54^ can be set by appropriate choice of the substrate surface plane. As such, the switching plane/IL formation and defined phase space for orthorhombic film growth provide new strategies toward the deterministic engineering of functional properties at the nanoscale.

## Methods

### Thin Film Growth

The LaVO_3_ films are grown on (101)_orth_ DyScO_3_ substrates by pulsed laser deposition using an excimer KrF laser run at 1 Hz repetition rate and at high pulse fluence (2 J/cm^2^). Deposition occurs on substrates heated from 800 to 900 °C (standard value of ∼880 °C) in a 5 × 10^–7^ mbar oxygen atmosphere from a ceramic target of LaVO_4_; cooling is performed under the same oxygen pressure. In situ reflection high energy electron diffraction reveals that the deposition evolves from a layer-by-layer growth mode during the first few unit cells to a mainly step-flow mode. Atomic force microscopy identifies the high surface quality of the films: the film topography for all the thicknesses displays a step-and-terrace structure, mirroring the (101)_orth_ DyScO_3_ substrate surface. For more details, see Meley.^22^

### X-ray Diffraction

The scans were acquired with a X’Pert PRO PanAlytical diffractometer equipped with a Ge(220) monochromator and a triple-axis analyzer.

### Scanning Transmission Electron Microscopy

Samples for STEM were prepared either by a combination of mechanical polishing using an Allied High Tech MultiTech polishing system, followed by argon ion beam milling with a Gatan PIPS II system to electron transparency, or by focused ion beam milling using a Zeiss NVision 40. All STEM imaging data were acquired using a monochromated, double aberration-corrected FEI (Thermo Fisher Scientific) Titan Themis 60-300 operated at a high tension of 300 kV and using a probe semiangle of convergence of 20.7 mrad and beam current of ∼40 pA. HAADF STEM images were acquired using a Fischione photomultiplier tube (PMT) detector. Unless otherwise stated, detector collection semiangles of ∼50–200 mrad were applied, using a nominal camera length of 115 mm. For quantitative analysis of atomic column positions, image stacks were recorded at 90° rotations between consecutive frames. These stacks then underwent rigid and nonrigid alignment using the SmartAlign software,^55^ in order to reduce artifacts from system noise and scan drift. In order to decouple scan distortions from the atomic column row directions, the images were acquired with an angle of ∼10–15° between the fast scan direction and one of the principal directions of the atomic rows. This methodology was successfully applied for frames up to 4k × 4k pixels in size. In the HAADF images, *A* and *B* cation positions were identified by fitting two-dimensional Gaussian functions using Atomap.^56^ From these measurements, quantified maps and averaged depth profiles of Δ*y* and Δ*z* AM displacements were calculated using custom Python scripts. The *A*-site ϵ_*yx*_ strain maps presented in Supporting Information Figures S10 and S11 were made using the methodology described in Meley et al.^21^ In the table of contents figure, we instead use adapted geometrical phase analysis.^15^

When ABF images were recorded to visualize O sites, these were acquired simultaneously to the HAADF image series with the same nominal 115 mm camera length, using a Gatan PMT detector mounted at the entrance of a Gatan GIF Quantum ERS, giving collection semiangles of ∼10.6–24.3 mrad. Scan distortions were corrected by first performing rigid and nonrigid alignment of the HAADF image stack, and then applying the HAADF-determined corrections to the simultaneously acquired ABF image stack. In this way, the rigid and nonrigid alignment is unaffected by distortions from residual aberrations, sample mis-tilts or improper defocus that can have a stronger effect on the phase-contrast ABF image than incoherent HAADF image. O column positions were similarly identified using Atomap, with *B*–O–*B* angle plots calculated using a custom Python script. Subframe averaging was performed with the SmartAlign Template Matching Module.^55^

Analytical data were acquired using the same instrument, with STEM-EELS maps recorded with the Gatan GIF Quantum ERS, and energy-dispersive X-ray spectroscopy (EDXS) data recorded using the FEI/Thermo Fisher Scientific ChemiSTEM 4 quadrant silicon drift detector system. The EDXS data were recorded using the same probe semiangle of convergence and HAADF detector set up as previously described. In contrast, the EELS data were recorded using a monochromated setup, with a probe semiangle of convergence of ∼18 mrad, spectrometer semiangle of collection of ∼36 mrad, and HAADF angles of ∼74–170 mrad. The STEM-EELS elemental maps were prepared using the analysis functions in Gatan DigitalMicrograph 3.5. Note that the V map of Figure 3 and Supporting Information Figure S3 was integrated only from the V *L*_3,2_ peaks, in order to avoid contribution from the O *K*-edge. STEM-EDXS chemical maps and line profiles were prepared using Thermo Fisher Scientific Velox 3.10 software, applying a Schreiber-Wims ionization cross-section model for elemental quantification.

PACBED patterns were acquired in two different ways. The PACBED data presented in the main text were acquired using the Gatan Ultrascan charge-coupled device camera of the GIF. The PACBED data presented in the Supporting Information were acquired using a MerlinEM (Quantum Detectors) Medipix3. For this latter measurement, the microscope was operated at 200 kV high tension. All the PACBED patterns shown are corrected for rotation with respect to their accompanying STEM images.

STEM image simulations were performed with Dr. Probe software,^57^ using the imaging conditions detailed above for experimental acquisition, with aberrations and defocus set to 0. A source size of 0.015 nm was applied. PACBED simulations were performed with μSTEM,^36^ using 4 × 4 nm supercells created with JEMS software;^58^ see Supporting Information Figure S7.

### Second-Principle Calculations

A second-principles model of CaTiO_3_ was used to perform structural relaxations with applied constraints, as described in the main text, mimicking the conditions exhibited by the LaVO_3_ thin film grown on the DyScO_3_ substrate. Relaxations were performed using supercell sizes up to 124 × 4 × 4 repetition of the 5-atom pc perovskite structure (9920 atoms). The atomic relaxations were performed using the Multibinit package,^59^ until the maximum force become smaller than 10^–4^ eV Å^–1^.

The second-principles model was built with the Multibinit package which implements the second-principles approach outlined in Wojdeł et al.^60^ and Escorihuela-Sayalero et al.^61^ This method relies on a Taylor expansion of the potential energy surface (PES) around the reference cubic structure in terms of all structural degrees of freedom, with coefficients then determined from first-principles data. In this scheme, the energy includes harmonic and anharmonic contributions in terms of individual atomic displacements, macroscopic strains and their couplings, with the long-range dipole–dipole interaction treated explicitly. At the harmonic level, the coefficients are exactly those directly computed from density functional perturbation theory. At the anharmonic level, the most relevant terms are selected and their coefficients are fitted in order to reproduce the energies, forces and stresses computed from DFT for a set of configurations properly sampling the PES.

The training set of first-principles DFT data contained more than 5000 structures, calculated with the Abinit software package, making used of a plane-wave pseudopotential approach.^59,62−64^ The DFT calculations were performed within the generalized gradient approximation making use of the Wu-Cohen parametrization,^65^ that was further checked^42^ to provide results totally comparable to PBEsol.^66^ The plane wave energy cutoff was of 40 Ha, and the Brillouin zone sampling equivalent to a 8 × 8 × 8 grid for the 5-atom perovskite cubic uc. The final effective atomic potential contains 360 polynomial terms, until order 8, and was further validated by comparison with first-principles data. It describes well the relative phase stability and distortion amplitudes of the most important metastable phases of CaTiO_3_. It accurately reproduces the phonon dispersion curves of its *Pnma* ground state and captures its temperature behavior. It further reproduces the atomic relaxation at ferroelastic twin walls, reproducing results previously obtained from first-principles by Barone et al.;^67^ see Supporting Information Figure S13. Exhaustive details on the construction of the second-principles model of CaTiO_3_ and of its validation by comparison to first-principles calculations, are provided by Schmitt.^40^

## Supporting Information Available

Strain states for LaVO_3_ grown on a (101)_orth_ DyScO_3_; projections of *Pnma* perovskite structure; STEM HAADF and ABF of LaVO_3_ film below critical thickness on substrate [010]_orth_; STEM EELS, EDXS, HAADF and ABF, PACBED of LaVO_3_ films above critical thickness; comparison of experimental and simulated 2-D PACBED patterns and PACBED radial distribution functions for bulk LaVO_3_ and substrate DyScO_3_; atomic-resolution HAADF STEM images and *A*-site ϵ_*yx*_ strain maps on [110]_pc_; X-ray diffraction of  half-order peak for LaVO_3_ thickness series; comparison of first- and second-principles calculations of CaTiO_3_ twin wall polarization; structure energy as a function of intermediate layer thickness for simulated film thickness series; STEM image/simulation comparison across switching plane for [100]_pc_ zone axis (PDF)

## Notes

We cite a preprint version of this paper: Alexander, D. T. L.; Meley, H.; Schmitt, M. M.; Mundet, B.; Ghosez, P.; Triscone, J.-M.; Gariglio, S. Engineering Symmetry Breaking Interfaces by Nanoscale Structural-Energetics in Orthorhombic Perovskite Thin Films. 2024, 2401.08798. arXiv. https://arxiv.org/abs/2401.08798 (accessed Feb. 4, 2025).
