# Supplementary material for: Engineering Symmetry Breaking Interfaces by Nanoscale Structural-Energetics in Orthorhombic Perovskite Thin Films
Source: ACS Nano. 2025 Mar 6;19(10):10126–37. doi: 10.1021/acsnano.4c17020 (PMC11924306; doi:10.1021/acsnano.4c17020)
Supplement: Supplementary file 1 — nn4c17020_si_001.pdf [file nn4c17020_si_001.pdf]

Supporting Information for: Engineering Symmetry Breaking Interfaces by Nanoscale Structural-Energetics in Orthorhombic Perovskite Thin Films

Duncan T.L. Alexander,[*,†] Hugo Meley,[‡] Michael Marcus Schmitt,[¶] Bernat Mundet,[‡,†] Jean-Marc Triscone,[‡] Philippe Ghosez,[¶] and Stefano Gariglio[*,‡]

†*Electron Spectrometry and Microscopy Laboratory (LSME), Institute of Physics (IPHYS), École Polytechnique Fédérale de Lausanne (EPFL), CH-1015 Lausanne, Switzerland*

‡*Department of Quantum Matter Physics, University of Geneva, CH-1211 Geneva, Switzerland*

¶*Theoretical Materials Physics, Q-MAT, Université de Liège, 4000 Liège, Belgium*

E-mail: duncan.alexander@epfl.ch; stefano.gariglio@unige.ch

Table S1: Biaxial epitaxial strain states for $LaVO_3$ grown on a $(101)_{orth}$ $DyScO_3$ substrate with the film's orthorhombic long-axis (b_{orth}) either in-plane ($b_{orth} \parallel b_{pc}$) or out-of-plane ($b_{orth} \parallel c_{pc}$), as calculated from bulk lattice constants for the two compounds. ϵ_{yy} is lower for b_{orth} out-of-plane, because the pseudocubic unit cell lattice parameter of $LaVO_3$ is shorter parallel to b_{orth} than for the other two pseudocubic axes (themselves defined by a_{orth} and c_{orth}). For this reason, considerations of macroscopic strain energy alone favor film growth with b_{orth} out-of-plane.

b_{orth} orientation	$LaVO_3$ on $DyScO_3$
$b_{orth} \parallel b_{pc}$	$\epsilon_{xx} = 0.49\%$<br>$\epsilon_{yy} = 0.71\%$<br>$\epsilon_{xy} = 0$
$b_{orth} \parallel c_{pc}$	$\epsilon_{xx} = 0.49\%$<br>$\epsilon_{yy} = 0.61\%$<br>$\epsilon_{xy} = 0$

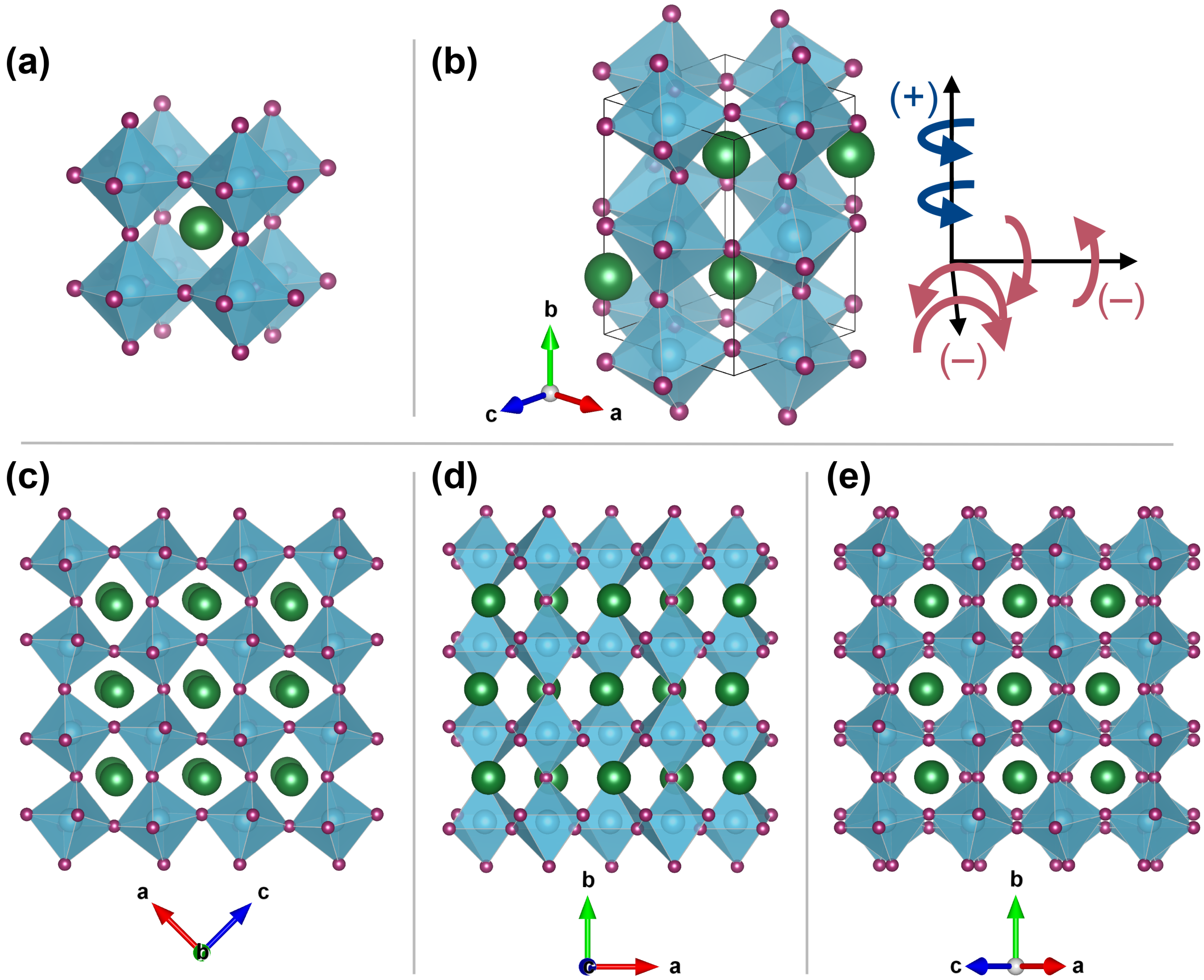

Figure S1: The ABO_3 orthorhombic perovskite structure with $Pnma$ space group. Panel (a) shows the basic cubic perovskite unit cell, with six oxygen anions (purple spheres) forming an octahedron centered around each corner-sited B cation (blue spheres). The A-site cation (green sphere) sits in the middle of four BO_6 octahedra. The unit cell of the orthorhombic perovskite structure is shown in (b). Using the standard $Pnma$ setting, this cell has lattice parameters $a_{\mathrm{orth}} \approx c_{\mathrm{orth}} \approx \sqrt{2}a_{\mathrm{pc}}$ and $b_{\mathrm{orth}} \approx 2a_{\mathrm{pc}}$ where a_{pc} is the lattice parameter of a reference 5-atom pseudocubic unit cell. The BO_6 octahedra of the orthorhombic lattice rotate in-phase along the b_{orth} axis and out-of-phase along the two pseudocubic axes that are in the $(010)_{\mathrm{orth}}$ plane (i.e. plane containing a_{orth} and c_{orth}); these rotation axes are shown schematically on the right. Projections along different axes highlight the various distortions of this orthorhombic structure: (c) the in-phase oxygen octahedra rotations (OOR) viewed along $[010]_{\mathrm{orth}}$; (d) the X_5^- mode antipolar motion of the A-site cations seen by looking down $[001]_{\mathrm{orth}}$ (the cations displace parallel to $[100]_{\mathrm{orth}}$); (e) the out-of-phase OOR seen along $[100]_{\mathrm{pc}}/[101]_{\mathrm{orth}}$, with this view also showing the X_5^- mode projected by a 45° angle. Structural models were prepared with the aid of VESTA,[1] using an $LaVO_3$ crystal structure file for panels (b–e).

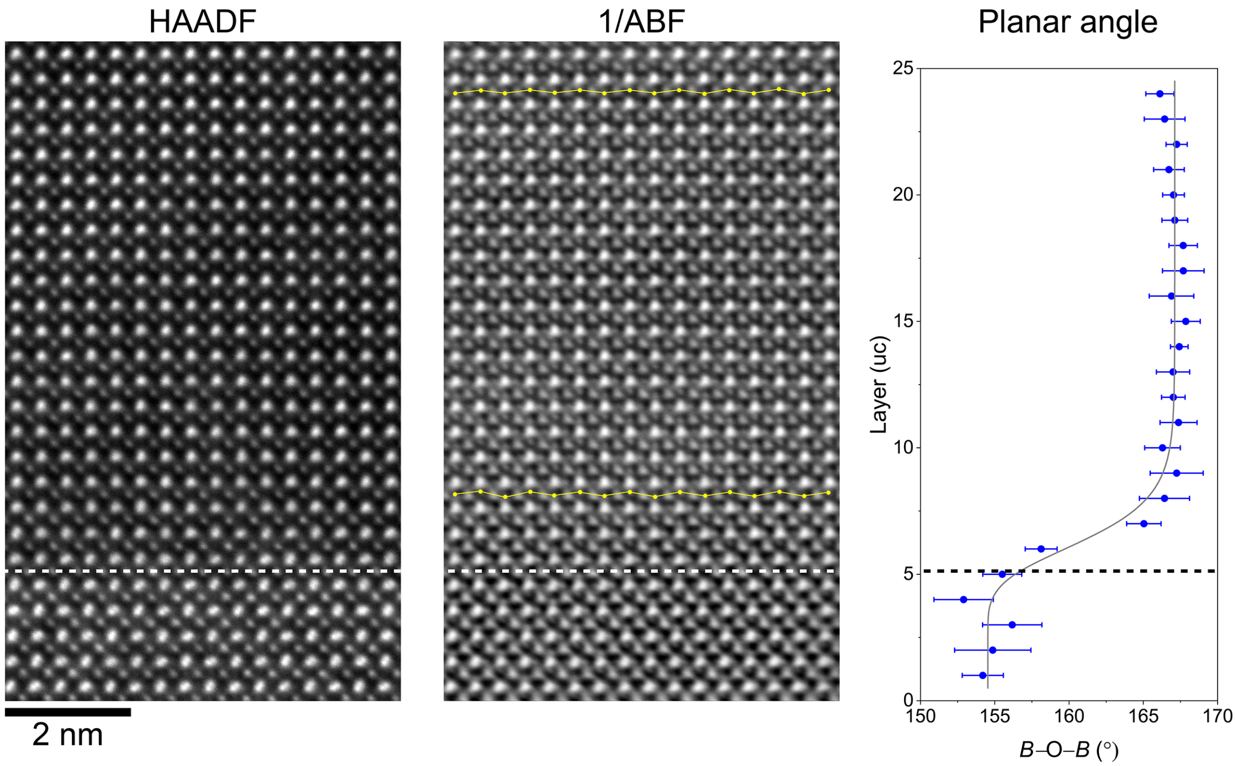

Figure S2: Simultaneously acquired HAADF and (inverted) ABF images of the first layers of a 52 uc $LaVO_3$ film grown on $DyScO_3$, recorded on the $[010]_{\mathrm{orth}}$ zone axis of the substrate in order to view the propagation of in-phase OOR from the $DyScO_3$ into the film. The film–substrate interface is indicated with the white dashed line. While data quality is suboptimal, particularly owing to a film–substrate mis-tilt from curvature of the FIB sample upon its preparation, O column positions show sufficient contrast for visualizing the propagation of in-phase OOR from the substrate across the whole film. This is for instance shown in yellow with hand-fitted planar O sites at the 3rd and 19th layers of the film. Atomap has been used to quantify the average planar B–O–B angle from layer to layer; the resulting data points on the right confirm the initial reduction in bond angle amplitude going into the film over some uc, followed by continuous propagation at roughly constant amplitude of the in-phase OOR. The results are consistent with the analysis of A-site AM presented in Figure 2. The black curve on this plot serves as a guide to the eye, while error bars correspond to the measurement standard deviation across each layer.

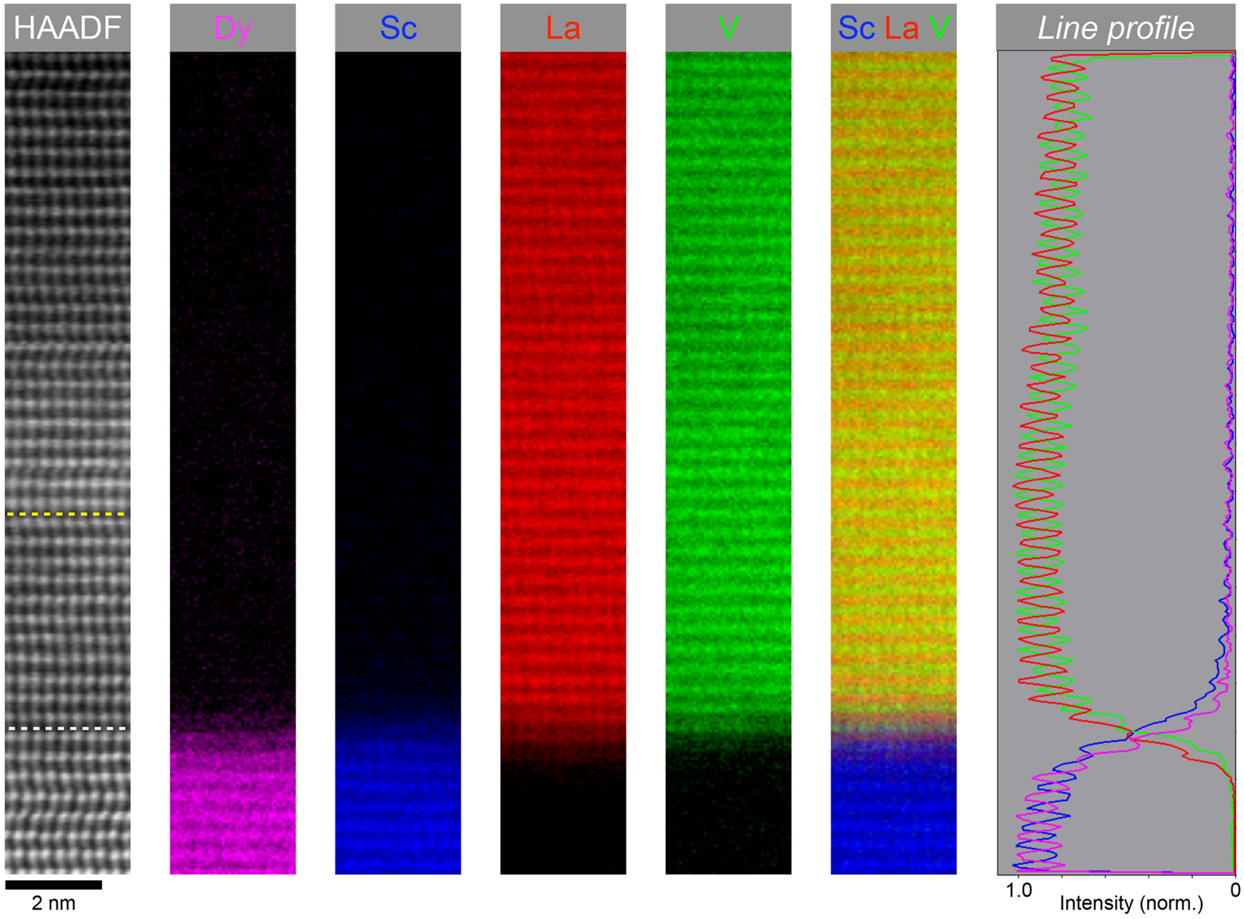

Figure S3: STEM-EELS mapping of $LaVO_3$ on $DyScO_3$ at the film–substrate interface of a film grown to thickness > 100 uc, imaged on the $[110]_{pc}$ zone axis. HAADF STEM image and associated Dy M, Sc L, La M and V L (quantified) maps and RGB composite map are shown. It is seen that the darker contrast in the IL of a HAADF image on this zone axis (indicated between white and yellow dashed lines) is not associated with a change in composition of the IL relative to the film bulk. This result is confirmed by the normalized elemental line profiles on the right, which also show that there are $\sim$2 uc of intermixing at the film–substrate interface. Higher in the film, the elemental line profiles decrease slightly in intensity; this time, the decrease is in accord with the HAADF intensity, indicating that the STEM lamella is reduced in thickness.

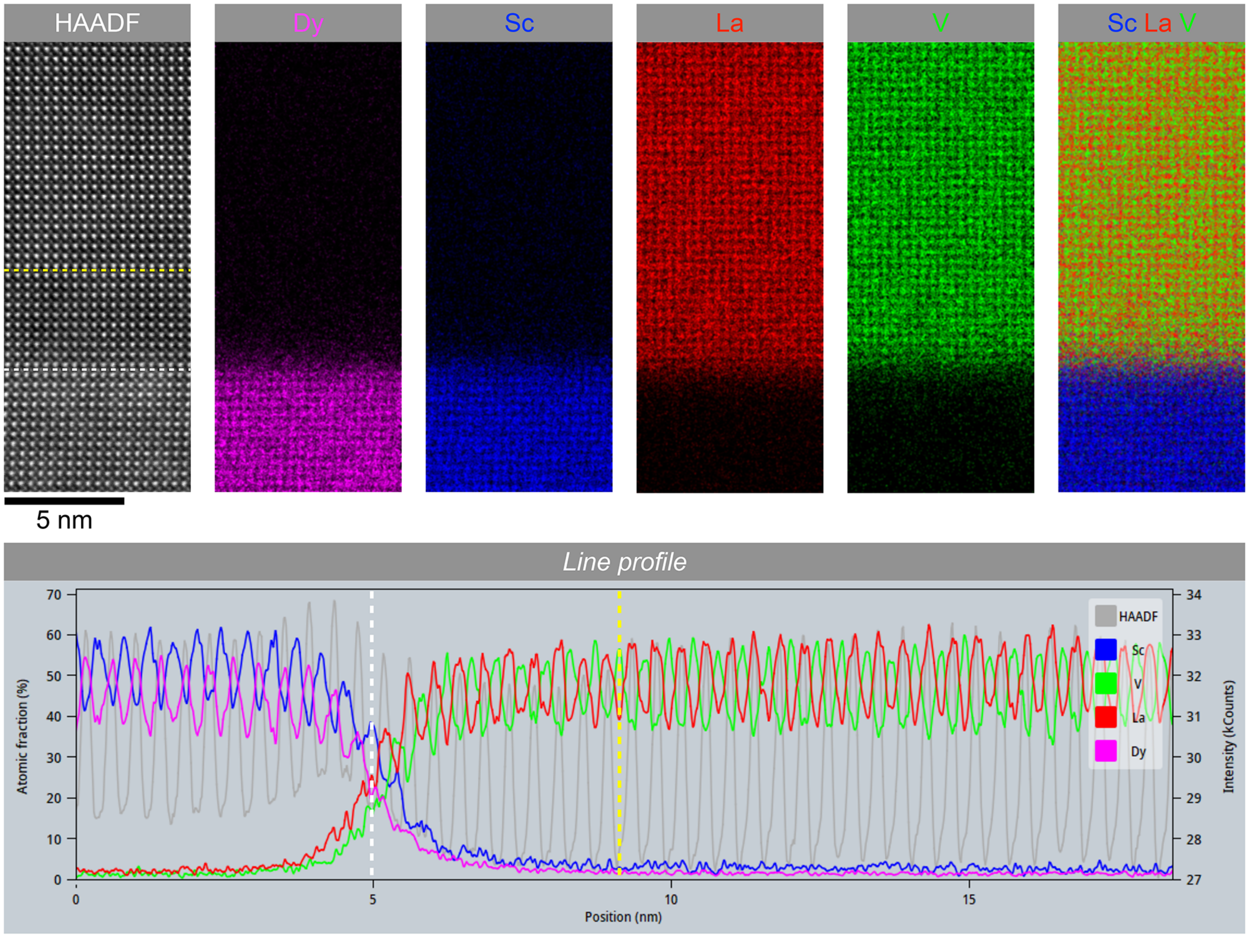

Figure S4: HAADF image with associated STEM-EDXS maps and integrated line profile of an 81 uc $LaVO_3$ film grown on $DyScO_3$, recorded at the film–substrate interface. The maps are recorded along the $[010]_{orth}$ zone axis of the substrate (i.e. the orthorhombic long axis). As for Supporting Information Figure S3, $\sim$2 uc of chemical intermixing is observed at the film–substrate interface. On this zone axis, the pseudocubic unit cell of the $DyScO_3$ shows a "shear" of $\sim 3°$ that is not present in the $LaVO_3$ (whose orthorhombic lattice parameters are nearly tetragonal in nature). The change in angle between the two gives an extra method for identifying the position of the film–substrate interface. This position—indicated with a white dashed line—correctly matches the chemically identified interface, which in turn agrees with the interface position identified from change in HAADF image contrast. (Note that, unlike the $[110]_{pc}$ zone axis, the HAADF contrast is not anomalous on this zone axis.) The top of the IL is indicated with a yellow dashed line, as established from the onset of X_5^- antipolar motion of the La cations as, at this position, the $LaVO_3$ zone axis changes from $[010]_{orth}$ in the IL to $[10\bar{1}]_{orth}$ in the film bulk. Atomic fractions in the line profile are calculated only with respect to the cations.

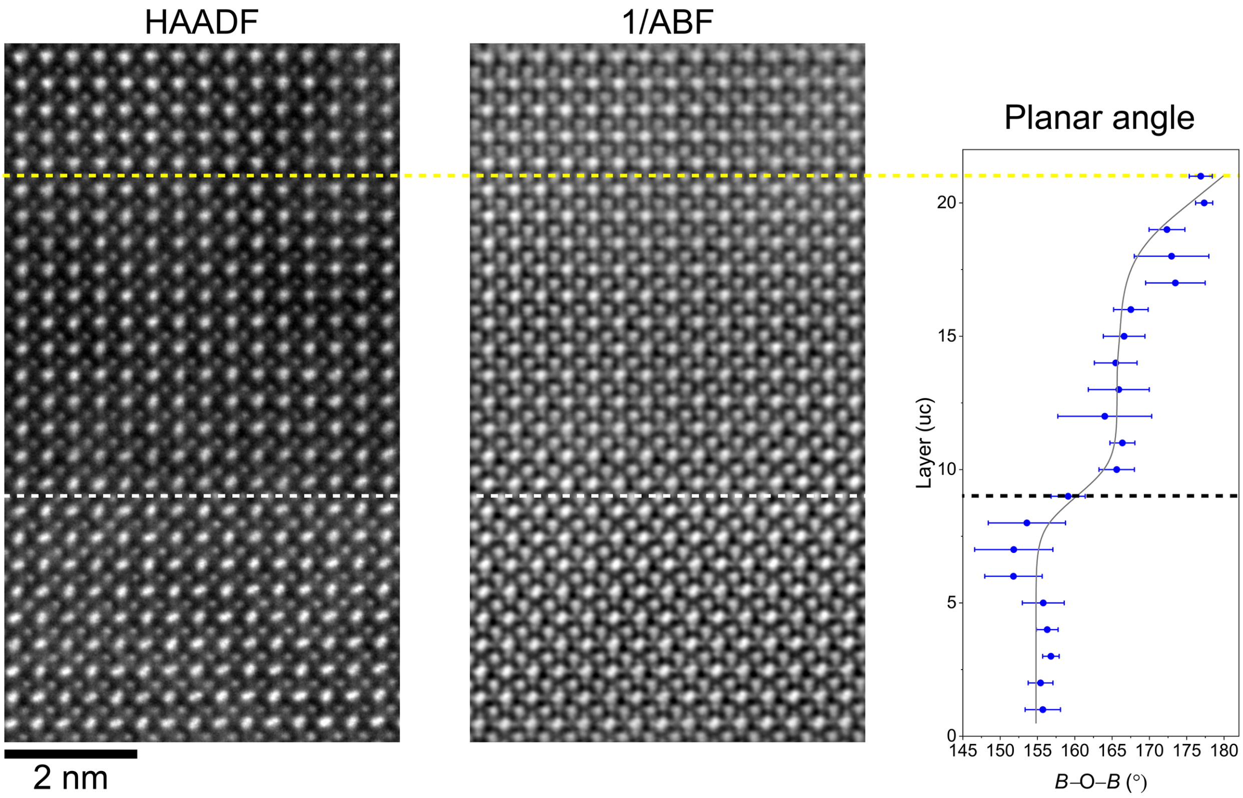

Figure S5: Simultaneously acquired HAADF and (inverted) ABF images of IL of 81 uc $LaVO_3$ grown on $DyScO_3$ recorded on the $[010]_{\mathrm{orth}}$ zone axis of the substrate, in order to view the propagation of in-phase OOR from the $DyScO_3$ into the IL. The film–substrate interface is indicated with the white dashed line, and the switching plane position with the yellow dashed line (as evaluated from the onset of X_5^- mode at the top). While data quality is suboptimal owing to experimental challenges—including a film–substrate mis-tilt from curvature of the FIB sample upon its preparation—the O column positions show sufficient contrast for visualizing the trend of B–O–B angles. Atomap has been used to quantify the average planar B–O–B angle from layer to layer. Results show the same trend as for the AM measurement in Figure 4: initial reduction of orthorhombic distortion (corresponding to an increase in bond angle amplitude) at the film–substrate interface indicated by the black dashed lines, followed by a plateau region at ∼165°, and then tending to a value of ∼180° at the top of the IL. The curve in black acts as a guide to the eye. Note that positions are not measured above the switching plane, because the planar O site becomes a projected dumbbell as the in-phase rotation axis switches to out-of-plane. In this region of the deposited film, the IL appears 1–2 uc thicker than the region shown in Figure 4. This is suggested to derive from a small instability in the formation energetics, that is discussed in the main text.

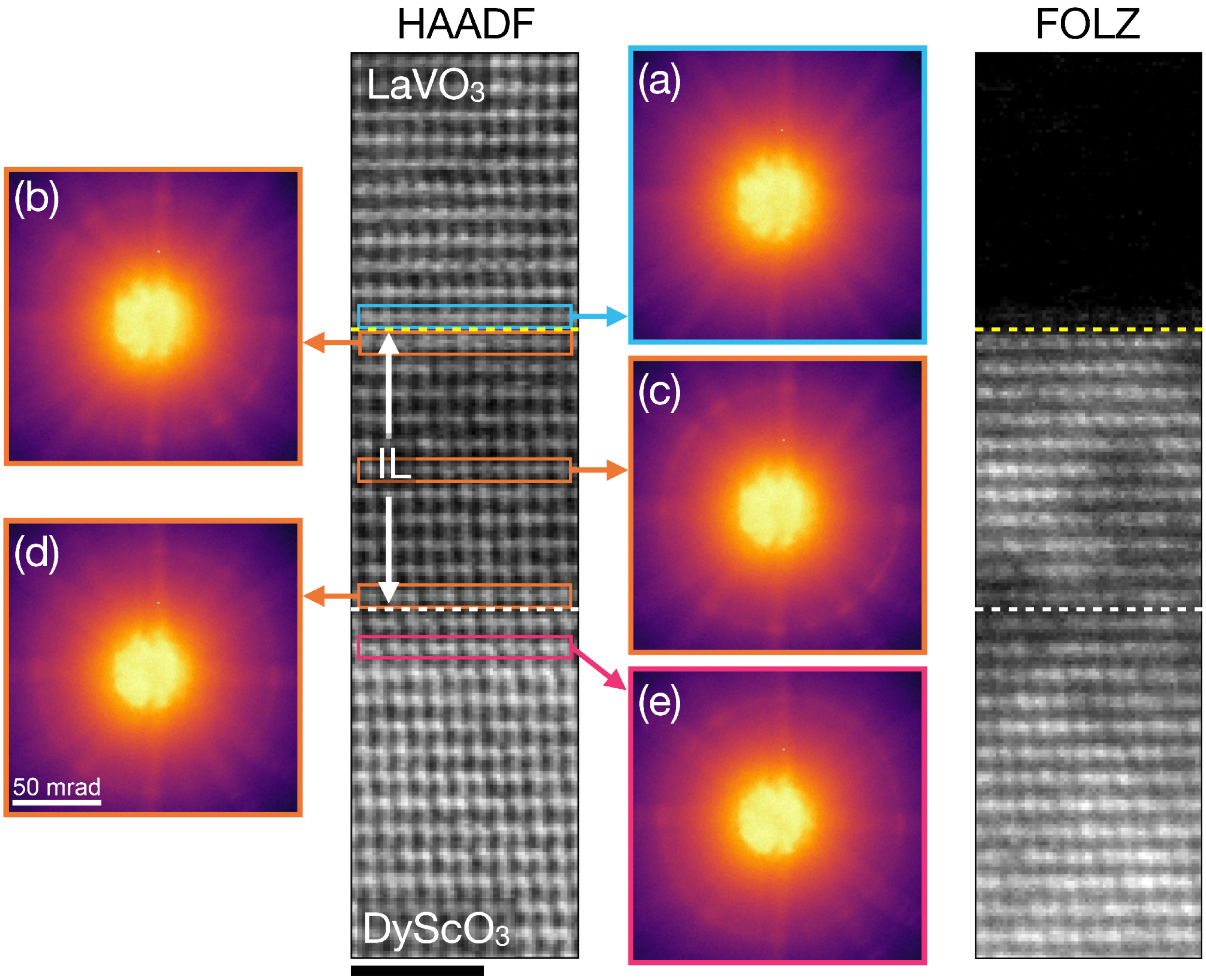

Figure S6: PACBED measurements of an 81 uc film of $LaVO_3$ grown on $DyScO_3$, recorded along the $[110]_{pc}/[11\bar{1}]_{orth}$ zone axis of the substrate. Each displayed pattern corresponds to the average STEM diffraction pattern from the associated 1 uc thick region shown on the HAADF STEM image. Patterns (b), (c) and (d) from the $LaVO_3$ IL maintain the characteristic low-angle first order Laue zone (FOLZ) of the $DyScO_3$ substrate that is seen in pattern (e). Stepping just one unit cell from (b) to (a) across the switching plane (indicated with a yellow dashed line) leads to a sharp loss of this FOLZ ring as the film discretely switches orientation from a $[11\bar{1}]_{orth}$ to a $[001]_{orth}$ zone axis. The data were acquired using a "4D-STEM" approach, recording a diffraction pattern at each probe position, with the microscope operated at a high tension of 200 kV (unlike 300 kV for the other measurements). The displayed HAADF image was generated by integrating the 4D-STEM signal over scattering angles from 80–140 mrad. In order to demonstrate the significance of the low-angle FOLZ, a FOLZ image on the right is generated from the FOLZ intensity by applying the methodology described in Nord et al.:[2] converting the 4D-STEM data to a radial distribution function (RDF), fitting and subtracting a background, and then fitting a Gaussian to the RDF peak from the FOLZ ring. Above the switching plane, the FOLZ image intensity drops sharply to zero because of the disappearance of the FOLZ ring. Scale bar: 2 nm.

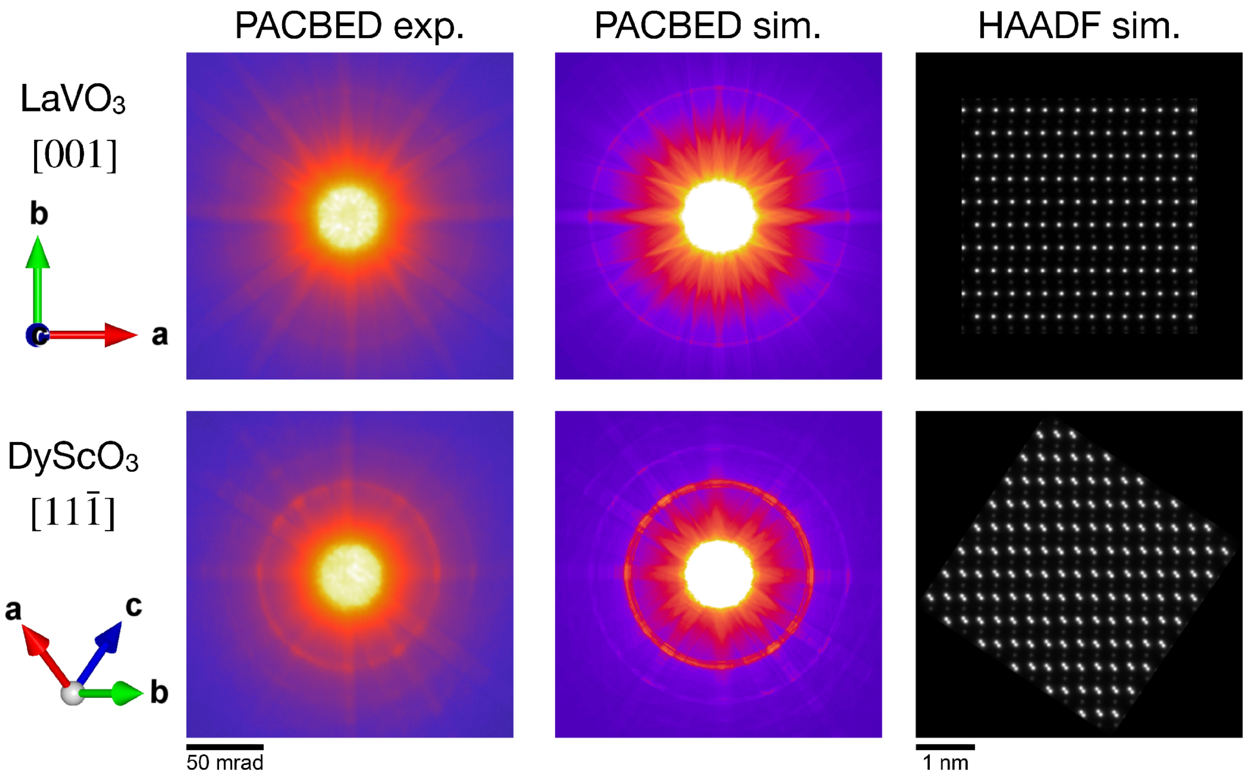

Figure S7: Comparison of experimental and simulated 2-dimensional PACBED patterns for $LaVO_3$ bulk film on $[001]_{orth}$ and $DyScO_3$ substrate on $[11\bar{1}]_{orth}$. The experimental patterns on the left are from Figure 3. The simulated patterns in the middle were made using μSTEM,[3,4] and show the full scattering (elastic plus thermal diffuse scattering). Each simulation utilized a 4×4 nm supercell; for illustration, the panels on the right show HAADF STEM simulations of the same supercells. The $DyScO_3$ PACBED and STEM simulations have been rotated to put the $(101)_{orth}$ substrate plane horizontal. Following recent work by Silinga et al.,[5] we can interpret that intensity peaks in the $DyScO_3$ FOLZ ring on a diagonal at about $-55°$ correlate to the X_5^- A-site antipolar displacements along a_{orth}, that projects along the same chord. These displacements further create the tilted Dy dumbbells in the simulated HAADF image on this zone axis, as visualized experimentally in Supporting Information Figures S10 and S11. Note that small distortions in the experimental PACBED patterns likely derive from parasitic reciprocal space aberrations from the image corrector of the double-corrected microscope used to acquire the data.[6,7]

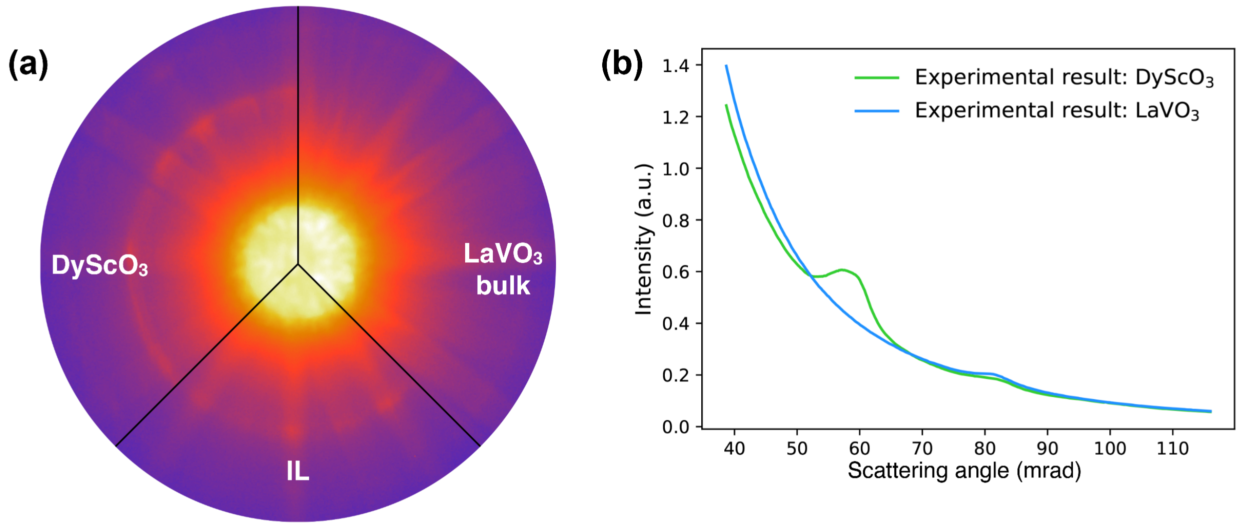

Figure S8: Anomalous HAADF intensity along the $[11\bar{1}]_{\mathrm{orth}}$ zone axis: experimental data. (a) PACBED patterns from Figure 3, arranged into a composite pattern for easy comparison. (b) From the $DyScO_3$ substrate and $LaVO_3$ film bulk PACBED patterns, experimental radial distribution functions (RDFs) of the signal intensity versus scattering angle are determined. The substrate, having a $[11\bar{1}]_{\mathrm{orth}}$ zone axis shows a strong peak at ∼60 mrad scattering angle, for a low angle FOLZ ring. This peak is not present in the film bulk, which instead has a $[001]_{\mathrm{orth}}$ zone axis. Outside of this peak, the $LaVO_3$ actually has a higher intensity than the substrate, even though it has a lower average atomic number (Z_{av}) of 19.6 *vs.* 21, that typically leads to decreased intensity from thermal diffuse scattering (intensity $\propto Z_{\mathrm{av}}^{1.6-2}$). It is suggested that, on the substrate's $[11\bar{1}]_{\mathrm{orth}}$ zone axis, strong coherent elastic scattering into the low-angle FOLZ leads to an anomalously low thermal diffuse scattering (TDS), from a partitioning effect on the total scattering.

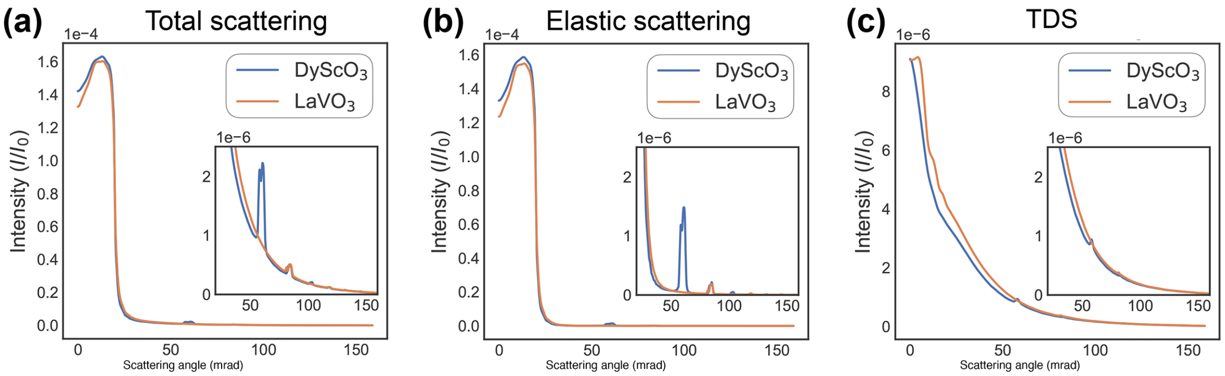

Figure S9: Anomalous HAADF intensity along the $[11\bar{1}]_{\mathrm{orth}}$ zone axis: RDF simulations. The quantum excitation of phonons model of the μSTEM simulation software is applied, allowing the incoherent inelastic scattering of phonon-driven TDS to be separated from coherent elastic scattering.[3,4,8] μSTEM was used to simulate PACBED patterns, from which RDFs were calculated. (a), (b) and (c) respectively show the total scattering, elastic scattering and TDS RDFs for substrate and bulk film. The total scattering simulations confirm that, outside of the FOLZ, the DyScO_3 has a lower scattering intensity than the LaVO_3, as seen experimentally in Supporting Information Figure S8. Moreover, the TDS curves confirm that this is specifically because the DyScO_3 has an anomalously low thermal diffuse scattering—lower than that for the LaVO_3, despite the higher Z_{av} of DyScO_3. Therefore, increased coherent elastic scattering into the FOLZ leads to a net reduction of phonon-driven TDS via a partitioning effect. This result in turn explains the darker HAADF contrast of the LaVO_3 IL compared to the film bulk, when the sample is imaged on the $[110]_{\mathrm{pc}}$ zone axis (Figure 3). The IL keeps the $[11\bar{1}]_{\mathrm{orth}}$ zone axis of the substrate. It therefore also suffers from an anomalously low thermal diffuse scattering. By using a HAADF inner collection angle that excludes the FOLZ, the HAADF signal from the IL is then lower in intensity than the bulk LaVO_3, giving rise to its darker contrast in the HAADF STEM image. For added insights, Supporting Information Figure S7 shows the full-scattering 2-dimensional PACBED patterns, compared to their experimental counterparts.

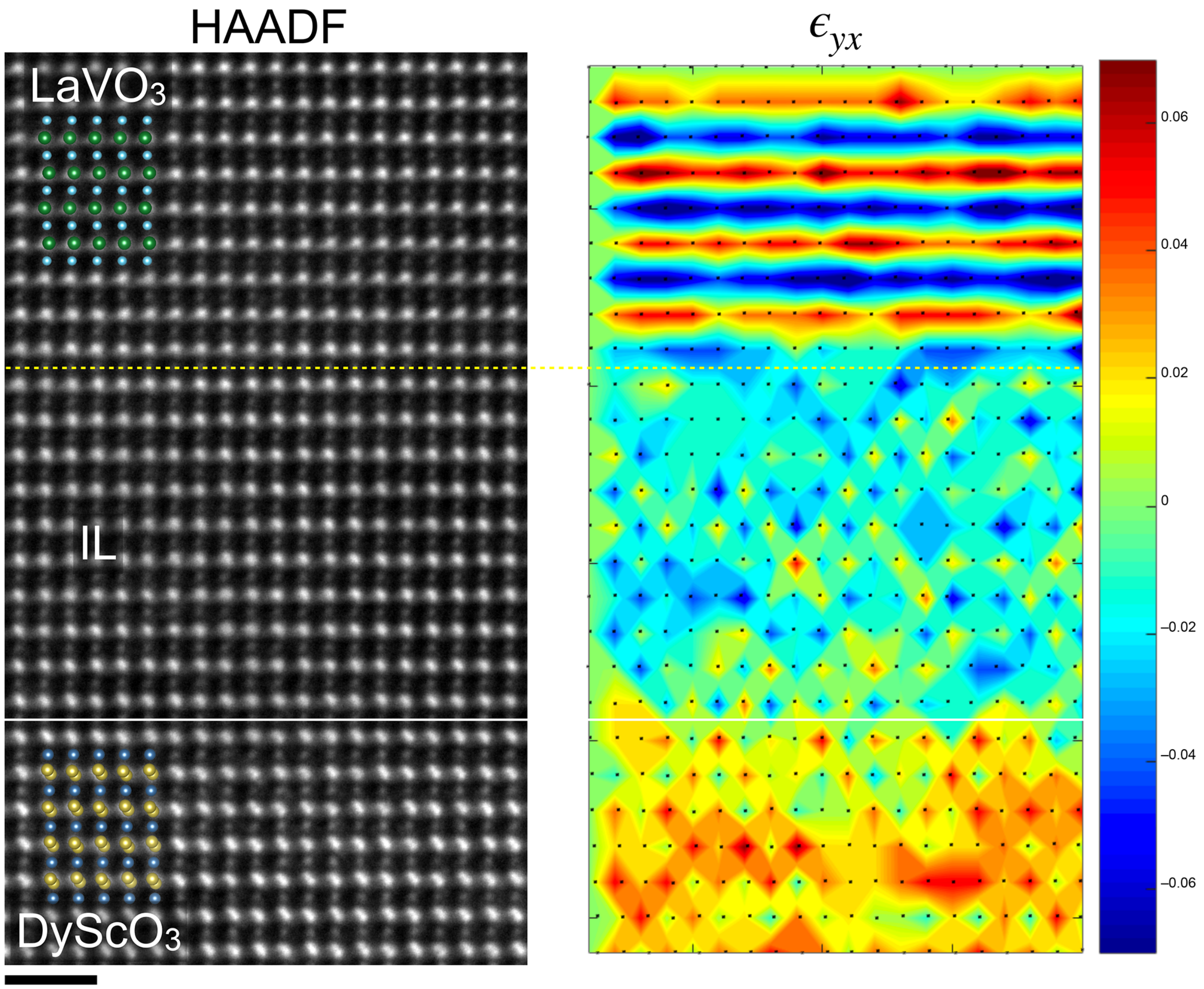

Figure S10: HAADF STEM image recorded on a $[110]_{pc}$ zone axis, corresponding to $[11\bar{1}]_{orth}$ for the $DyScO_3$ substrate. As explained in Supporting Information Figure S7, on this axis the X_5^- AM produces tilted Dy dumbbells, whose 0.6 Å separation is just resolved in the image. This dumbbell form propagates through the IL as elliptical La columns, and then stops at the switching plane when the film orientation switches to b_{orth} out-of-plane and takes a $[001]_{orth}$ zone axis. Given this zone axis, the bulk $LaVO_3$ shows a pure X_5^- AM, as successive horizontal La planes displace to the left and right. This displacement is highlighted by a variant of adapted geometrical phase analysis,[9] where we make an ϵ_{yx} strain tensor map based on A-site column locations.[10] This renders the X_5^- AM as positive and negative streaks in the strain map. In the $DyScO_3$ and IL, the ϵ_{yx} strain map instead shows a checkerboard pattern of diamonds, that derives from the R_4^- AM of in–out A-site displacements parallel to c_{orth}. Note that the tilt of the near-vertical planes in the $DyScO_3$ results from $a_{orth} \neq c_{orth}$. In comparison, $a_{orth} \approx c_{orth}$ in $LaVO_3$. Scale bar: 1 nm.

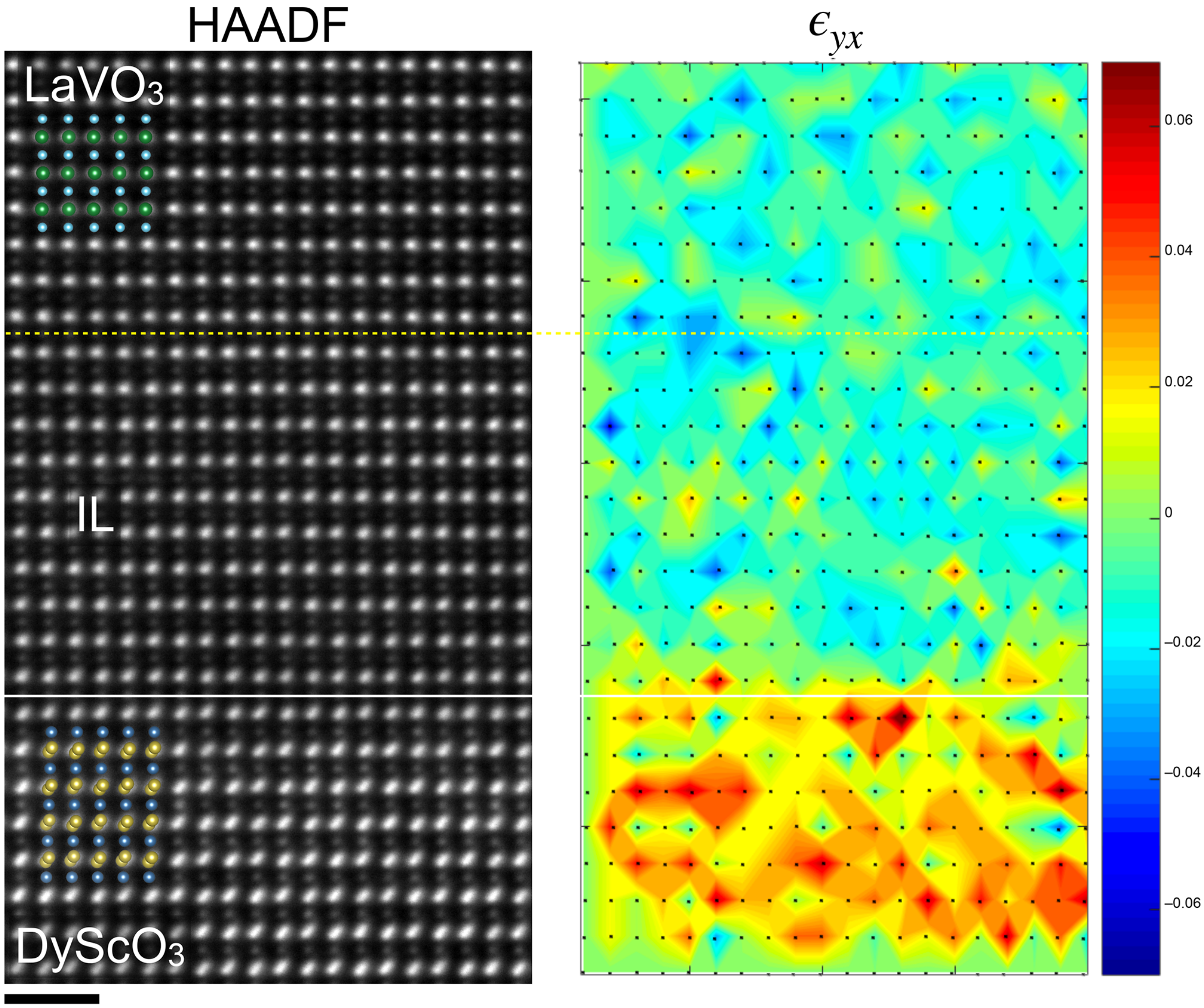

Figure S11: HAADF STEM image recorded on a $[110]_{pc}$ zone axis, corresponding to $[\bar{1}\bar{1}1]_{orth}$ for the $DyScO_3$ substrate. (The inverse zone axis of Supporting Information Figure S10.) The bulk $LaVO_3$ is oriented on a $[100]_{orth}$ zone axis. Because of this, it shows a pure—but very subtle—R_4^- AM, as successive pairs of La columns move closer together/further apart along one horizontal plane. The La displacements of one plane are in anti-phase with the plane above or below it. This R_4^- AM is rendered as a checkerboard diamond pattern in the ϵ_{yx} strain tensor map based on A-site column locations. Since the $DyScO_3$ and IL also show an R_4^- character, from c_{orth} being projected at an angle on their $[\bar{1}\bar{1}1]_{orth}$ zone axis (see Supporting Information Figure S7), the switch from IL to $LaVO_3$ bulk is not obvious. It would, however, be clear using PACBED, similarly to Figure 3 and Supporting Information Figure S6. Scale bar: 1 nm.

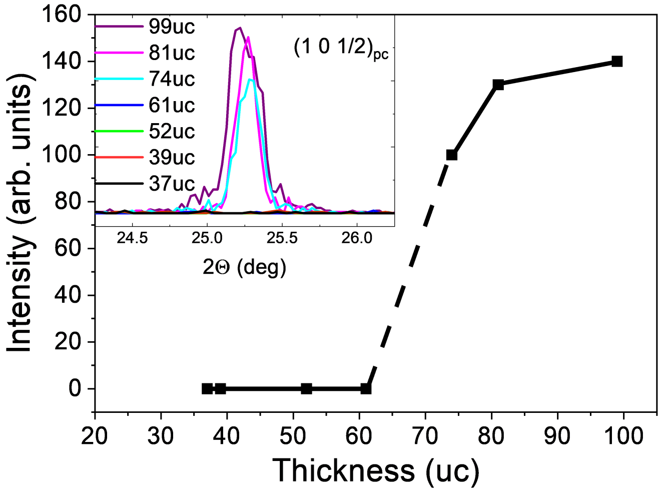

Figure S12: The measurement by XRD of the $(1\,0\,\frac{1}{2})_{\mathrm{pc}}$ half-order peak (see inset) reveals the presence of the in-phase (+) OOR axis (b_{orth}) in the out-of-plane direction. The intensity of this reflection becomes non-zero only for $LaVO_3$ films with a thickness above a critical value of 62–74 uc.

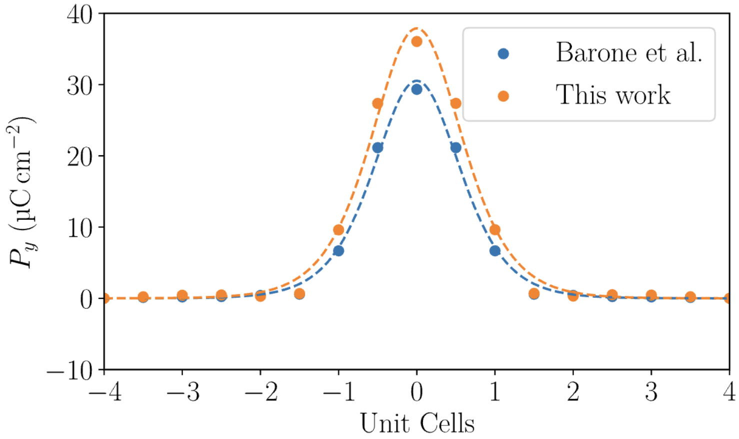

Figure S13: Comparison of the evolution of the polarization at a twin wall of $CaTiO_3$ as obtained from our second-principles model and from first-principles calculations by Barone et al.; see Figure 2 of Ref.[11]

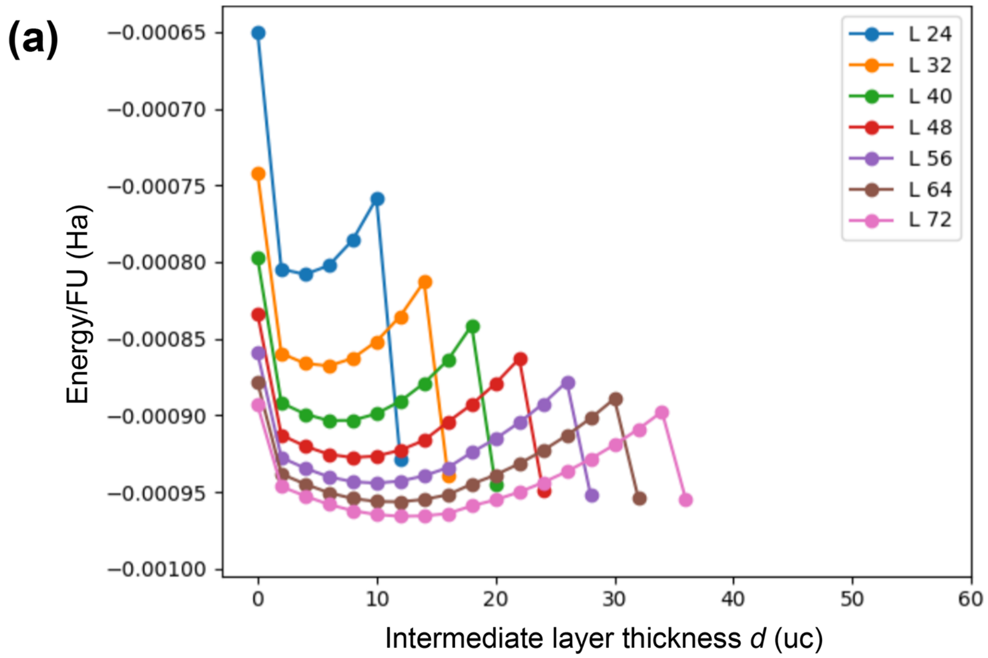

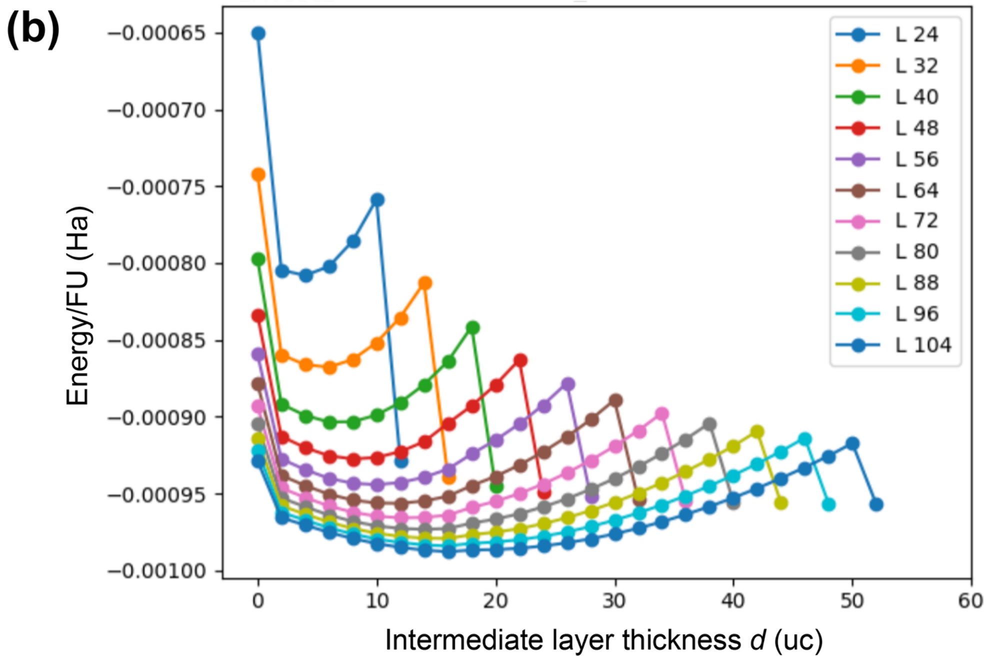

Figure S14: From the second-principles calculations, structure energy as a function of IL thickness d for supercells ranging in uc dimension from (a) $L = 24$ to $L = 72$ and (b) $L = 24$ to $L = 104$.

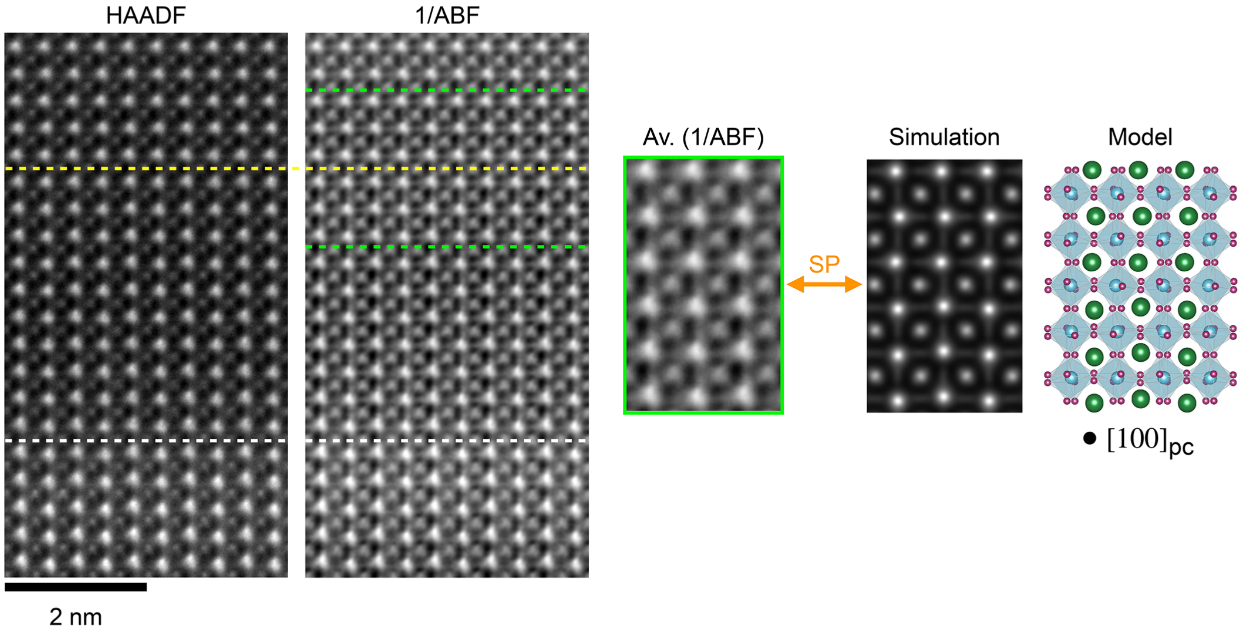

Figure S15: On the left, simultaneously acquired HAADF and (inverted) ABF images of IL of 81 uc $LaVO_3$ grown on $DyScO_3$, recorded on the $[100]_{pc}/[10\bar{1}]_{orth}$ zone axis of the substrate. The ABF image has been treated with a Wiener filter to reduce noise. The white and yellow dashed lines respectively indicate the film–substrate interface and the switching plane. From the full 20 uc width of the initial ABF image, subframe averaging using the SmartAlign Template Matching Module has been applied.[12] From the averaged image, the segment around the switching plane is shown in the middle "Av. (1/ABF)" panel, whose vertical positioning corresponds to the green dashed lines on the initial 1/ABF image. This experimental image is compared to a simulated image based on the second-principles modeling-determined structure on the right. The streaks between the A-sites derive from the O dumbbells that themselves are indicative of the out-of-phase OOR on this $[100]_{pc}$ zone axis. In the model, it is seen that this OOR mode propagates across the switching plane (SP) with negligible damping. By comparing the experimental image to the simulated one, it is seen that the experimental image is consistent with this finding.

References

1. Momma, K.; Izumi, F. *VESTA3* for Three-Dimensional Visualization of Crystal, Volumetric and Morphology Data. *J. Appl. Crystallogr.* **2011**, *44*, 1272–1276.

2. Nord, M.; Ross, A.; McGrouther, D.; Barthel, J.; Moreau, M.; Hallsteinsen, I.; Tybell, T.; MacLaren, I. Three-Dimensional Subnanoscale Imaging of Unit Cell Doubling Due to Octahedral Tilting and Cation Modulation in Strained Perovskite Thin Films. *Phys. Rev. Mater.* **2019**, *3*, 063605.

3. Allen, L. J.; D'Alfonso, A. J.; Findlay, S. D. Modelling the Inelastic Scattering of Fast Electrons. *Ultramicroscopy* **2015**, *151*, 11–22.

4. Allen, L. J.; Brown, H. G.; D'Alfonso, A. J.; Findlay, S.; Forbes, B. D. MuSTEM Software (V5.2). 2018; `https://github.com/HamishGBrown/MuSTEM`.

5. Silinga, A.; Allen, C. S.; Barthel, J.; Ophus, C.; MacLaren, I. Measurement of Atomic Modulation Direction Using the Azimuthal Variation of First-Order Laue Zone Electron Diffraction. *Microsc. Microanal.* **2023**, *29*, 1682–1687.

6. Krause, F. F.; Schowalter, M.; Grieb, T.; Müller-Caspary, K.; Mehrtens, T.; Rosenauer, A. Effects of Instrument Imperfections on Quantitative Scanning Transmission Electron Microscopy. *Ultramicroscopy* **2016**, *161*, 146–160.

7. Jones, L. Quantitative ADF STEM: Acquisition, Analysis and Interpretation. *IOP Conf. Ser. Mater. Sci. Eng.* **2016**, *109*, 012008.

8. Forbes, B. D.; Martin, A. V.; Findlay, S. D.; D'Alfonso, A. J.; Allen, L. J. Quantum Mechanical Model for Phonon Excitation in Electron Diffraction and Imaging Using a Born-Oppenheimer Approximation. *Phys. Rev. B* **2010**, *82*, 104103.

9. Mundet, B.; Hadjimichael, M.; Fowlie, J.; Korosec, L.; Varbaro, L.; Domínguez, C.; Triscone, J.-M.; Alexander, D. T. L. Mapping Orthorhombic Domains with Geometrical Phase Analysis in Rare-Earth Nickelate Heterostructures. *APL Mater.* **2024**, *12*, 031124.

10. Meley, H.; Karandeep; Oberson, L.; de Bruijckere, J.; Alexander, D. T. L.; Triscone, J.-M.; Ghosez, P.; Gariglio, S. Structural Analysis of $LaVO_3$ Thin Films Under Epitaxial Strain. *APL Mater.* **2018**, *6*, 046102.

11. Barone, P.; Di Sante, D.; Picozzi, S. Improper Origin of Polar Displacements at $CaTiO_3$ and $CaMnO_3$ Twin Walls. *Phys. Rev. B* **2014**, *89*, 144104.

12. Jones, L.; Yang, H.; Pennycook, T. J.; Marshall, M. S. J.; Van Aert, S.; Browning, N. D.; Castell, M. R.; Nellist, P. D. Smart Align—A New Tool for Robust Non-Rigid Registration of Scanning Microscope Data. *Adv. Struct. Chem. Imaging* **2015**, *1*, 8.
